# The Uve1 Endonuclease Is Regulated by the White Collar Complex to Protect *Cryptococcus neoformans* from UV Damage

**DOI:** 10.1371/journal.pgen.1003769

**Published:** 2013-09-05

**Authors:** Surbhi Verma, Alexander Idnurm

**Affiliations:** Division of Cell Biology and Biophysics, School of Biological Sciences, University of Missouri-Kansas City, Kansas City, Missouri, United States of America; University of California San Francisco, United States of America

## Abstract

The pathogenic fungus *Cryptococcus neoformans* uses the Bwc1-Bwc2 photoreceptor complex to regulate mating in response to light, virulence and ultraviolet radiation tolerance. How the complex controls these functions is unclear. Here, we identify and characterize a gene in *Cryptococcus*, *UVE1*, whose mutation leads to a UV hypersensitive phenotype. The homologous gene in fission yeast *Schizosaccharomyces pombe* encodes an apurinic/apyrimidinic endonuclease acting in the UVDE-dependent excision repair (UVER) pathway. *C. neoformans UVE1* complements a *S. pombe uvde* knockout strain. *UVE1* is photoregulated in a Bwc1-dependent manner in *Cryptococcus*, and in *Neurospora crassa* and *Phycomyces blakesleeanus* that are species that represent two other major lineages in the fungi. Overexpression of *UVE1* in *bwc1* mutants rescues their UV sensitivity phenotype and gel mobility shift experiments show binding of Bwc2 to the *UVE1* promoter, indicating that *UVE1* is a direct downstream target for the Bwc1-Bwc2 complex. Uve1-GFP fusions localize to the mitochondria. Repair of UV-induced damage to the mitochondria is delayed in the *uve1* mutant strain. Thus, in *C. neoformans UVE1* is a key gene regulated in response to light that is responsible for tolerance to UV stress for protection of the mitochondrial genome.

## Author Summary

The majority of fungi sense light using the White Collar complex (WCC), a two-protein combination of a photoreceptor and a transcription factor. The WCC regulates circadian rhythms, sexual development, sporulation, metabolism, and virulence. As such, the exposure to light controls properties of fungi that are beneficial and detrimental to people, depending on the species and its interaction with humans. Despite the importance of light on fungal biology, the underlying evolutionary benefit of light-sensing in fungi has remained a mystery. Here we identify a DNA damage repair endonuclease, Uve1, required for UV stress tolerance in the human pathogen *Cryptococcus neoformans*. *UVE1* is a direct target of the WCC in *C. neoformans*, and *UVE1* homologs are also regulated by WCC in two other major lineages of fungi, the Ascomycota and Mucoromycotina. The divergence of the three groups indicates that for about a billion years the same transcription factor complex has regulated a common gene to protect fungal genomes from deleterious effects of light. Curiously, in *C. neoformans* Uve1 localizes to mitochondria and contributes to mitochondrial DNA repair, implicating its importance in genome repair of this organelle. Thus, light-sensing in fungi exists to protect them against harmful light, and likely all other responses to light relate to or are a secondary consequence of this selective pressure.

## Introduction

The ability to sense light provides well-known advantages to organisms, such as adapting to photosynthetic light sources in plants and for vision in animals, yet the benefits of light-sensing in non-photosynthetic and non-motile organisms are less established. The fungi contain a suite of potential photoreceptor proteins, with the White Collar complex (WCC) being found throughout the fungal kingdom, except for species in which the two genes encoding the complex were lost. Light influences different responses in different fungi, including phototropism, induction of pigmentation, asexual and sexual sporulation, changes is primary and secondary metabolism, and regulating the circadian clock: most of which are controlled, where established, by the WCC [1]–[3]. A major question is what advantage is provided in using light as an environmental signal to regulate these processes. One compelling hypothesis is that protecting DNA from damage provides a selective pressure, and that wavelengths in the visible spectrum are sensed to indicate the presence of deleterious ultraviolet radiation. However, a DNA repair system common to light-sensing fungi and that acts directly downstream of the WCC is unknown to date.

The White collar-1 and White collar-2 proteins interact to form a complex (WCC) capable of sensing blue and near UV light. Both proteins were originally characterized in the ascomycete fungus *Neurospora crassa* [4]–[7] where WC-1 acts as a photoreceptor. In *N. crassa*, WCC has roles to play both in light and dark environments. In the dark the WCC has a major function as a circadian clock component, via regulation of *FRQ* gene expression [8]–[10]. Light-dependent functions of WCC include conidiation, carotenoid production and mating [11], [12]. The photons and the signal are transduced via the chromophore flavin adenine dinucleotide (FAD) bound within an N-terminal specialized type of PAS domain (from the Per, Arnt, Sim proteins), named the LOV (light, oxygen and voltage) domain [13]. The two proteins interact using other PAS domains, to form the transcription factor complex that regulates transcription of target genes via their GATA-type zinc finger DNA-binding domains [4], [5], [7], [14].

The human pathogen *Cryptococcus neoformans* is a member of the phylum Basidiomycota, a distant relative to *N. crassa*. The fungus grows vegetatively as a budding yeast and during mating in a dikaryotic filamentous form. *C. neoformans* is divided into two varieties: var. *grubii* is the most prevalent in the clinic and var. *neoformans* is less common but was the most amenable to experimental methods until the discovery of an opposite mating partner for var. *grubii* about a decade ago [15]. *C. neoformans* primarily causes disease in immunocompromised people. The closest relative to *C. neoformans* is *C. gattii*, which is also a human pathogen but with a greater tendency to infect immunocompetent individuals [16]. Homologs of *N. crassa wc-1* and *wc-2* are present in *Cryptococcus* species, designated *BWC1*/*CWC1* and *BWC2*/*CWC2*, and have been characterized in strains of both var. *grubii* and var. *neoformans* [17], [18]. The Bwc1-Bwc2 complex has three known functions in *C. neoformans*: it represses mating in the light, promotes virulence, and provides protection against UV light. The downstream targets of Bwc1-Bwc2 that control these functions remain to be elucidated.

Several genes have been identified from *C. neoformans* that are regulated by light. These include *SXI1*α and *MF*α*1*, found within the mating type locus and encoding a transcription factor and pre-pheromone protein required for sexual reproduction [17]. Their expression could explain the repression of mating by light. However, the regulation of these transcripts was compared after a long time exposure of 24 h in the light or constant darkness, such that this time point likely reflects indirect regulation by Bwc1-Bwc2. More recently a microarray experiment was carried out in *C. neoformans* to detect light-regulated transcripts with an hour of exposure to light. The *HEM15* gene, encoding ferrochetalase that is the last step in the heme biosynthetic pathway, was identified as a gene under control of Bwc1-Bwc2 [19]. However, the phenotype of the knockout *hem15* strain differs significantly from mutation of *BWC1* or *BWC2*. For example, *HEM15* is essential for viability, and yet the *bwc1* and *bwc2* mutants do not show any growth defects in the light or dark. Another light-regulated gene is *CFT1*, required for iron uptake and virulence [20], yet no iron-dependent phenotype of *bwc1* and *bwc2* mutants is known. Thus, while *CFT1* and *HEM15* may be targets of the Bwc1-Bwc2 complex, they likely play minimal roles in the physiological response of *C. neoformans* to light. Moreover these two genes are also under the control of other transcription factors in addition to WCC. As a transcription factor complex, it was puzzling that more light-regulated genes were not identified by microarray analysis that could potentially explain the phenotypes of deleting the WCC from *C. neoformans*.

The microarray results of *C. neoformans* contrast to ascomycete species. For instance, in *N. crassa* a microarray study in wild type, *wc-1*Δ, and *wc-2*Δ strains at different time periods identified 314 light-regulated genes, constituting 5.6% of the total detectable transcripts in the genome [11]. These genes were grouped into two broad categories, the early or late light responsive genes (ELRGs and LLRGs). Some of these ELRGs are involved in the synthesis of vitamins, photo-protective pigments, prosthetic groups and cofactors, cellular signaling, DNA processing, circadian rhythm and secondary metabolism. Many of the LLRGs are implicated in carbohydrate metabolism, fatty acid oxidation and free radical detoxification. In *N. crassa* there is also a link between the WCC and DNA repair, for example through the clock gene *prd-4*, which is a cell cycle checkpoint kinase 2 [21]–[23].

As the White Collar complex acts as an UV/blue light photoreceptor and mutation of the complex causes an increase in sensitivity to UV light, potential targets are hypothesized to be genes involved in repairing DNA damage for survival under UV stress. The *UVE1* gene of *C. neoformans* was previously identified in a UV sensitive strain in a collection of insertional mutants [24]. The product of *UVE1* is a homolog of an apurinic/apyrimidinic endonuclease that is best characterized in fission yeast *Schizosaccharomyces pombe*. In *S. pombe* the gene was called *UVDE* for UV damage endonuclease, and renamed *uve1* for consistency with nomenclature [25], [26]. The *mus-18*/*UVE-1* gene is the homolog characterized from *N. crassa* [27]. The protein removes UV-induced cyclobutane pyrimidine dimers and 6-4 photoproducts, acting in its own pathway termed the UVDE-dependent excision repair (UVER) pathway. UVDE recognizes single-stranded DNA nicks, apurinic/apyrimidinic sites, and nucleotide mismatches [26], [28]–[30], a suite of DNA lesions that also extends a possible role for UVDE in repairing the equivalent types of DNA damage caused by reactive oxygen species [31].

In *S. pombe*, UVDE is localized and functional in both the nucleus and mitochondria, and was suggested to act as a reserve mechanism for repairing UV-induced DNA damage in the mitochondria [32]. Homologs of *UVE1* are present in a subset of species in the Archaea, Bacteria and Eukaryotes [25], [33]–[37], with one exception being humans where there is no homolog. A preliminary northern blot experiment suggested that *UVE1* in *C. neoformans* var. *neoformans* is a light-regulated gene with two isoforms, triggering this investigation. We hypothesized that *UVE1* is a downstream target of the WCC that functions in repairing UV-induced damage, and tested this hypothesis in the experiments described below.

## Results

### *UVE1* is essential in *C. neoformans* for survival under UV stress

**Figure 1:**
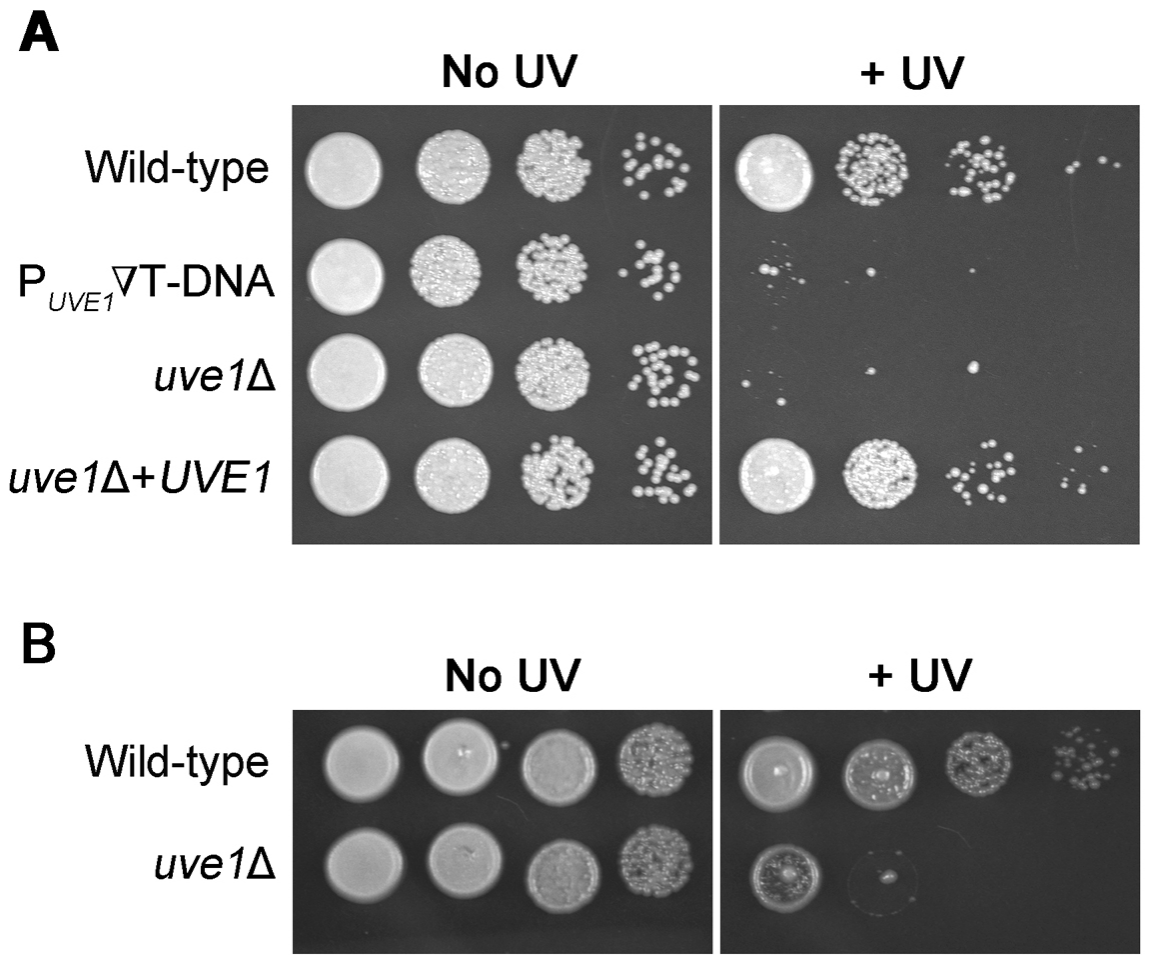
*UVE1* of *C. neoformans* confers resistance to UV stress. Ten-fold serial dilutions for different *Cryptococcus* strains grown at 30°C for 2 days. (A) Left panel untreated control and right panel treated with UV dose of 120 J/m^2^. Order of *C. neoformans* var. *grubii* strains from top to bottom KN99α, ST239E6, AI191, AI198. (B) Left panel untreated control and right panel treated with UV dose of 120 J/m^2^. Order of *C. neoformans* var. *neoformans* strains from top to bottom JEC21, AISVCN101.

A T-DNA insertion mutant within the promoter of the *UVE1* gene was identified previously [14]. Under UV stress conditions the mutant showed negligible survival as compared to the wild type (KN99α) and the unexposed strains (Figure 1A). Transformations derived from *Agrobacterium* T-DNA delivery can have phenotypes that are not due to the insertion of the T-DNA into the host genome. The UV sensitivity phenotype of the original mutant was verified by constructing *UVE1* gene replacement strains for both *C. n.* var. *neoformans* and *C. n.* var. *grubii*. The knockout strains in both varieties had reduced survival after exposure to UV (Figure 1A,B). To confirm that the UV sensitivity phenotype was because of the absence of *UVE1*, an *uve1*Δ strain was complemented with a wild type copy of *UVE1*. The *UVE1* complemented strain completely rescued the UV sensitivity phenotype (Figure 1). These results show that in *C. neoformans* the *UVE1* gene is required for survival under UV stress.

The responses of the *uve1* mutant to stresses other than UV light were tested, showing no other phenotypes (Figure S1). The gene also plays no major role in the formation of mating filaments (Figure S2A, B). A prior analysis, using comparative growth in a pool of 48 strains in the mouse lung, suggested the *UVE1* gene has no role in virulence [38]. To examine the *uve1*Δ strain in isolation, its virulence was tested in an insect model. While the *bwc1*Δ strain is less pathogenic than wild type in this model, the *uve1*Δ strain is equally virulent as wild type (Figure S2C). Thus, the only established function of Uve1 in *C. neoformans* is in response to UV stress.

### *UVE1* is a photoregulated gene in *C. neoformans*

**Figure 2:**
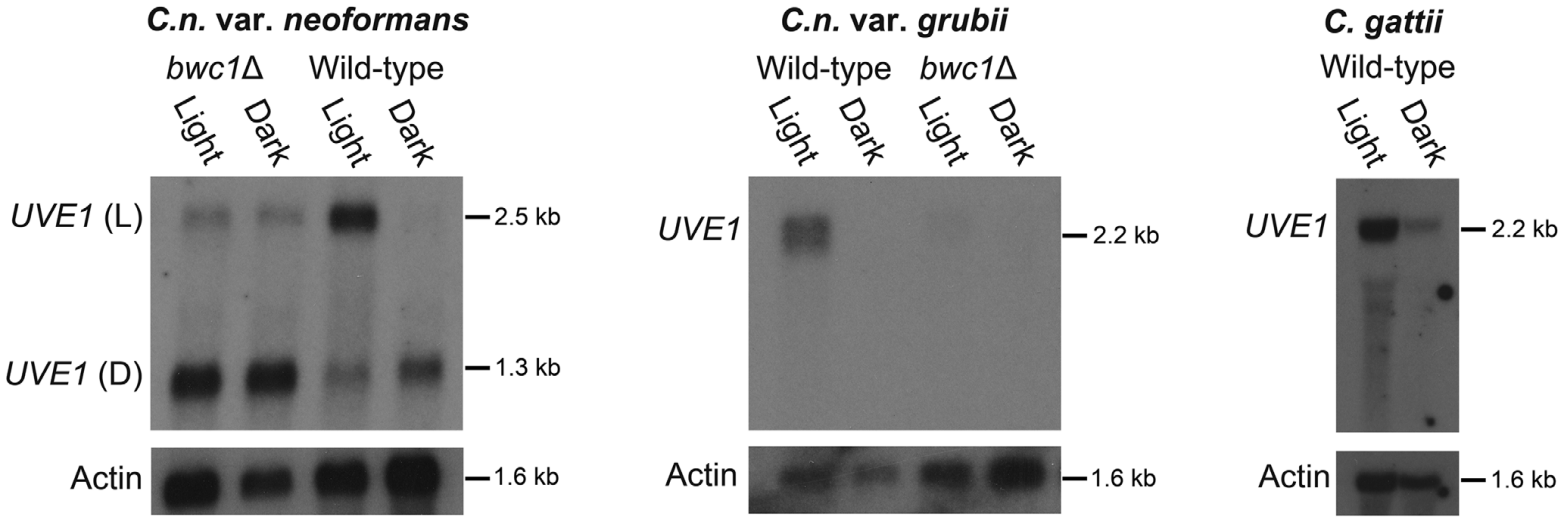
*UVE1* is a Bwc1-regulated gene in *Cryptococcus*. Northern blots of *C. n.* var. *neoformans*, *C. n.* var. *grubii* and *C. gattii*. From left to right, panel 1 is for JEC21 (WT) and AI5 (*bwc1*); the upper band is for *UVE1* light (L) isoform and the lower band is for *UVE1* dark (D) isoform. Panel 2 is for KN99α (WT) and AI81 (*bwc1*), panel 3 is for R265 (WT). All experiments were either 23 h dark+1 h light, (Light) or 24 h constant darkness (Dark). Blots were stripped and reprobed with actin as a loading control.

Since *UVE1* is required for surviving exposure to UV light, regulation of *UVE1* was tested in response to light. Wild type strains of *C. n.* var. *neoformans*, *C. n.* var. *grubii* and *C. gattii* were grown in the dark, and one replicate provided a one hour exposure to white light. Expression of the *UVE1* gene was examined by northern blot analysis on polyA RNA purified from these cultures. The *UVE1* transcript levels were higher in light grown conditions for all wild type strains (Figure 2). Two transcripts were observed for var. *neoformans* strain JEC21, one longer (L, for light) induced specifically under light conditions and another shorter (D, dark) expressed in the dark. For var. *grubii* and *C. gattii* one isoform of *UVE1* was expressed in response to light with negligible expression in dark grown conditions.

The role for the Bwc1 photoreceptor in *UVE1* induction by light was examined in the *bwc1*Δ deletion strains of *C. neoformans*. In the var. *neoformans* deletion there was loss of induction of the longer isoform by light, and residual expression of this isoform in both light and darkness. There was no observed effect of *bwc1*Δ on the shorter isoform in dark grown conditions. Dark isoforms expressed equally well in both light and dark conditions in some replicates, possibly reflecting the age of the culture or shading by other cells. In the var. *grubii bwc1*Δ mutant, the *UVE1* transcript was barely detectable under either illumination regime (Figure 2). These analyses in the *bwc1* mutant backgrounds indicate that *UVE1* expression is controlled by the Bwc1-Bwc2 complex.

**Figure 3:**
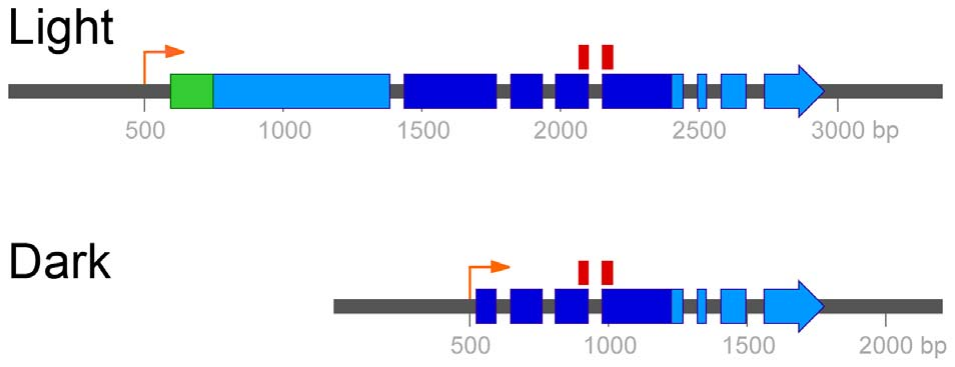
Two mRNA isoforms of *UVE1* are produced in *C. neoformans* var. *neoformans*. Boxes indicate coding regions, with the long light-induced isoform encoding a 660 amino acid residue protein and the shorter dark-expressed isoform encoding a 291 amino acid residue protein. Dark blue encompasses the pfam03851 domain that represents the conserved and active site of the endonuclease. The position of the 70 mer probe used in the *C. neoformans* microarray, which spans two exons common to both isoforms, is indicated above this region. The green region encodes the predicted mitochondrial localization signal. The orange arrows indicate the start of the *UVE1* light and dark transcripts.

Rapid amplification of cDNA ends (RACE) was used in the var. *neoformans* strain to define the 5′ and 3′ ends of the two transcripts that were produced in the light and the dark. The *UVE1* dark isoform is part of the *UVE1* light isoform, with the dark isoform starting in the middle of the light isoform and both sharing a common 3′ end (GenBank accessions KF234405 and KF234406; Figure 3). Alignment of the Uve1 homologs from various fungi indicated that the dark isoform of *C. neoformans* is truncated and missing key residues in the active site of this protein (Figure 3; Figure S3).

The light-dependent regulation of *UVE1* in wild type strains suggested that induction of the gene prior to UV exposure would correlate with increased UV resistance. The wild type, *bwc1*Δ and the complemented strains were grown in complete darkness overnight. One set was kept in the dark and the other set exposed to light for 2 hours, prior to treatment of both sets with UV light (Figure S4). All three strains grown in darkness showed similar levels of sensitivity to UV light. Exposure of the strains with the wild type copy of *BWC1* to light before UV stress increased their resistance to UV, a property not seen for the *bwc1*Δ strain. Hence, light signaled by Bwc1 promotes UV resistance.

Two isoforms of *UVE1* were observed in the var. *neoformans* strain, raising the possibility that differential transcript sizes may be a common feature for DNA repair genes in *C. neoformans*. To identify other genes involved in protecting the fungus against UV damage, a collection of 1200 defined knock out mutants in the var. *grubii* background [38] was screened for those sensitive to UV light. 13 strains were identified, including the *uve1*Δ strain in the collection (Figure S5A). For the corresponding genes, northern analysis were performed for var. *neoformans* and var. *grubii* cultured under light and dark conditions (Figure S5B). No altered size or induction in response to light was observed, as had been for the *UVE1* gene.

### Photoinduction of *UVE1* homologs is conserved in fungi

**Figure 4:**
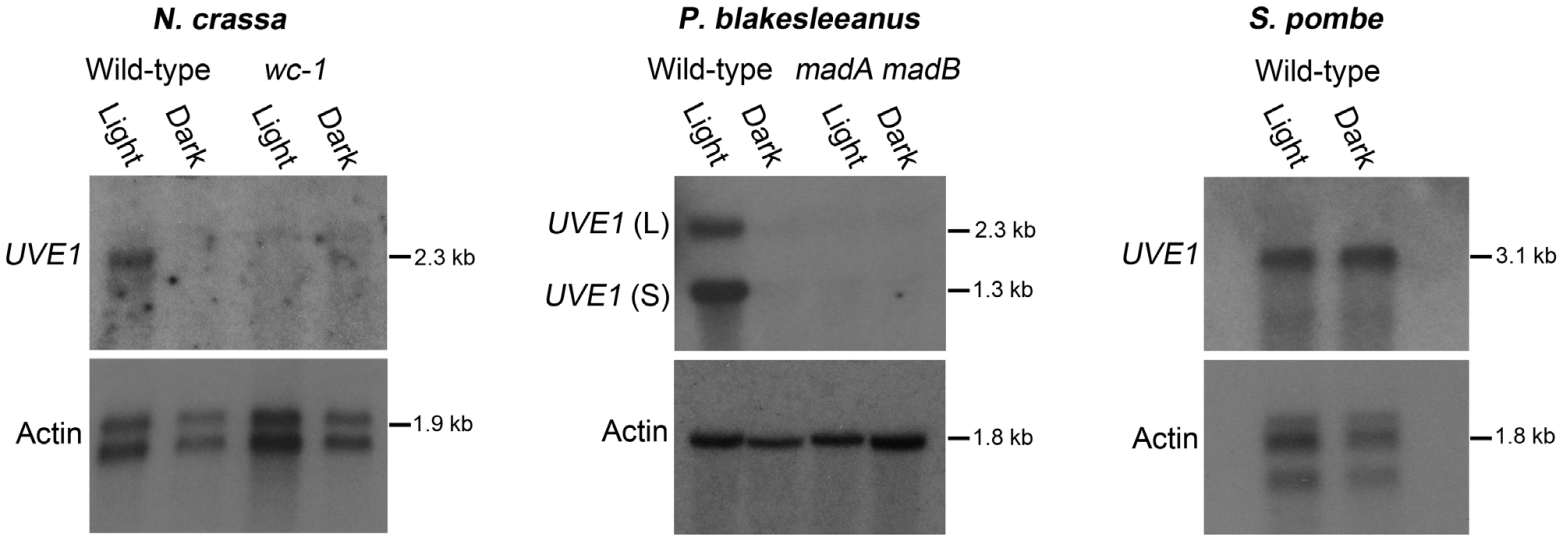
Light regulation of *UVE1* homologs through the White Collar complex is conserved in fungi. Northern blots for *UVE1* homologs from *N. crassa* [FGSC 4200 (WT), FGSC 4398 (*wc-1*)], *P. blakesleeanus* [NRRL1555 (WT), L51 (*madA madB*)], and *S. pombe* [L972 (WT)]. All experiments were 1 h light exposure (Light) or constant darkness (Dark). Blots were stripped and reprobed with actin as a loading control. For clarity, the gene name *UVE1* is used to refer to all homologs (*mus-18 N. crassa*; *uvdE P. blakesleeanus*; *uve1 S. pombe*).

To examine if *UVE1* photoregulation is common among fungi, northern blot analysis of *UVE1* was performed in two fungi, *Neurospora crassa* and *Phycomyces blakesleeanus* (Figure 4). The species are light-sensing models in the phylum Ascomycota and subphylum Mucoromycotina, respectively. In *N. crassa* the expression of *UVE1* has already been reported in a microarray study of the light-induced genes [11]. For the *N. crassa* wild type strain we observed by northern blot analysis the induction of one transcript in light grown conditions and minimal expression of the *UVE1* transcript in dark grown mycelia, confirming the previous microarray data. For the *P. blakesleeanus* wild type strain two transcripts were present in light grown conditions that were both absent in samples grown in the dark. Based on the expressed sequence tag information in the genome database, the 5′ end of the *UVE1* homolog is shared between the two transcripts and the 3′ end differs, the reverse of the situation in *C. neoformans* var. *neoformans*. The longer isoform in *P. blakesleeanus* has a 3′ extension due to transcriptional read-through into the 3′ neighboring gene.

We also examined the transcript profile of *UVE1* in a *white collar-1* mutant (*wc-1*) of *N. crassa* and a *madA-madB* mutant of *P. blakesleeanus* (*madA* and *madB* are functional *wc-1* and *wc-2* homologs in this species [39]). We observed complete loss of light-dependent induction of *UVE1* in these mutant strains of *N. crassa* and *P. blakesleeanus*. As an additional control to show that *UVE1* induction was not an indirect effect of light on the media, *UVE1* expression was examined in *S. pombe*, a “blind” species because it encodes no homologs of the WCC. We observed equal transcript levels of the *S. pombe UVE1* homolog in cultures grown under light and dark conditions (Figure 4). These studies demonstrate that *UVE1* is photoregulated among highly-diverged fungal species, and substantiates the *WC-1* dependent light-induction of *UVE1* in the fungal kingdom.

### Cellular localization of *C. neoformans* Uve1 light and dark isoforms

**Figure 5:**
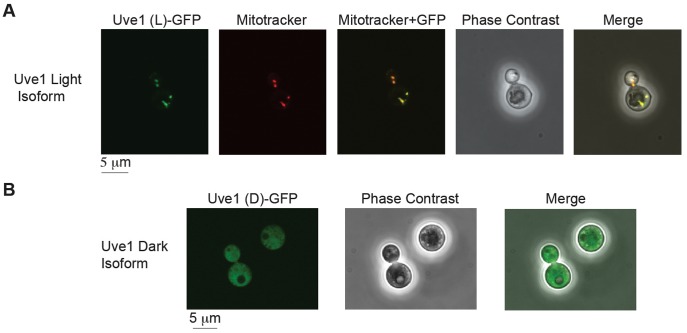
Subcellular localization of Uve1 (L)-GFP and Uve1 (D)-GFP from *C. neoformans* var. *neoformans* in the vegetative yeast cells of *C. neoformans* var. *grubii* (A) Uve1 (L)-GFP localization and (B) Uve1 (D)-GFP localization.

The protein sequences predicted for the two isoforms in var. *neoformans* from RACE were examined by bioinformatic approaches for their subcellular localization. PSORT II and MitoProt analysis of *UVE1* light and dark isoforms predicts the longer form to be most likely mitochondrial and no specific localization pattern for the dark isoform. To confirm these predictions, light and dark isoforms of *UVE1* were fused to the N-terminal end of GFP and expressed in the *uve1*Δ strain AI191. To assess mitochondrial localization we used MitoTracker red, which specifically stains respiring mitochondria. Confocal fluorescence microscopy for the GFP-fused light form of Uve1 showed co-localization of Uve1-GFP with MitoTracker red, giving a yellow fluorescence in merged images (Figure 5A). No expression of Uve1-GFP light form was observed in the nucleus, confirmed by co-staining with Hoechst (Figure S6). For the dark isoform of Uve1-GFP, GFP localization was throughout the cell, but clearly excluded from the mitochondria (Figure 5B; Figure S6). Transformation of the *UVE1-GFP* constructs into a *uve1*Δ genetic background enabled a test of their functionality in complementing the UV sensitive phenotype. Expression of the light form of *UVE1* rescued in part the UV sensitive phenotype of strain AI191; however, the dark isoform fused to GFP did not. These experiments suggest that the light isoform of *UVE1* is localized solely to the mitochondria, and as such it protects the mitochondrial genome from lethal effects of UV-induced DNA damage in *C. neoformans*.

### Functional conservation exists between Uve1 homologs of the basidiomycete *C. neoformans* and the ascomycete *S. pombe*

**Figure 6:**
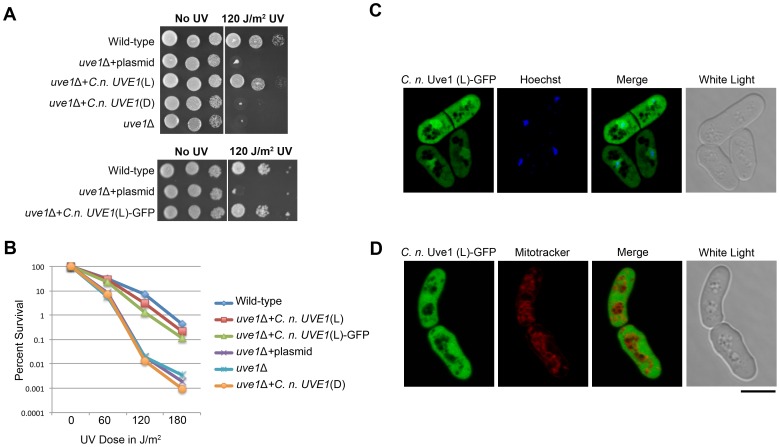
*C. neoformans* Uve1 is functionally similar to *S. pombe* UVDE. (A) The *C. neoformans* light (L) and dark (D) isoforms of *UVE1* were expressed in an *uve1* deletion strain of *S. pombe*. (A) Ten-fold serial dilutions for different strains of *S. pombe* grown at 30°C for 2 days. Left panel untreated control and right panel treated with UV dose of 120 J/m^2^. The strains used, from top to bottom are L972, AISVSP2, AISVSP4, AISVSP3 and AISVSP1. For the lower portion of the figure strains used are L972, AISVSP2, AISVSP15. (B) Graph of survival of strains L972, AISVSP4, AISVSP15, AISVSP2, AISVSP1 and AISVSP3 in response to UV stress of 0, 60, 120 and 180 J/m^2^. (C) Subcellular localization of *C. neoformans* Uve1 (L)-GFP compared to nuclei (Hoechst) in strain AISVSP1. (D) Localization of Uve1 (L)-GFP compared to mitochondria (MitoTracker). Scale bar = 10 µm.

The function of Uve1 in the fungi at a biochemical level is best characterized in *S. pombe* [25], [29], [40], [41]. Alignment of the two homologs suggests that they are similar, sharing residues within the active site of the enzyme (Figure S3). To infer functional similarity, a cross-species complementation test was performed. An *uve1*Δ knockout was generated in *S. pombe* by replacing the gene via homologous recombination with the KanMX cassette that confers resistance to G-418. We then expressed in this *S. pombe* knockout strain the cDNA clones of light or dark isoforms of *UVE1* from *C. neoformans* var. *neoformans*. The UV sensitivity of the *S. pombe* strains was tested (Figure 6A, B). The *uve1* deletion strain was highly sensitive to UV irradiation, as was the control strain transformed with the empty vector. In the strain expressing the light isoform of *UVE1*, UV sensitivity was rescued as the strain survived UV doses equivalent to the wild type strain. For the *uve1::KanMX*+*C. neoformans* dark isoform, no rescue in the UV sensitive phenotype was observed. These observations suggest that the *C. neoformans* light isoform of *UVE1* is functionally active and has the equivalent biochemical functions of Uvde from *S. pombe* required for repair of DNA damaged by UV exposure. It also suggests that the dark isoform of *C. neoformans* Uve1 may not have any function in conferring protection against UV.

### Cellular localization of *C. neoformans* Uve1 in *S. pombe*

*C. n*. var *neoformans* Uve1 was localized in *S. pombe* as a GFP fusion to see if the rescue of UV sensitivity phenotype in *S. pombe* is due to complementing mitochondrial or nuclear genome repair by Uve1 (Figure 6C, D). The Uve1-GFP construct was functional, complementing the UV sensitive phenotype of the *S. pombe uve1* mutation (Figure 6A, B). The localization of Uve1 (L)-GFP is in part nuclear, as confirmed by co-localization with the nuclear Hoechst stain (Figure 6C). No localization in mitochondria was observed (Figure 6D). These results suggest that the *Cryptococcus* Uve1 protein, which seems to be important for mitochondrial DNA repair in *C. neoformans*, also plays a role in nuclear DNA repair in *S. pombe*. This observed localization pattern conforms to previous reports where Uve1 in *S. pombe* repairs nuclear DNA after UV stress, rather than mitochondrial DNA [32].

### The *C. neoformans uve1* mutant is impaired in its repair of mitochondrial DNA

If Uve1 localizes to mitochondria in *C. neoformans*, it is expected to play a role in mitochondrial DNA repair in consequence of DNA damage due to UV stress. As no nuclear localization was observed for Uve1, a negligible role of this endonuclease is expected in nuclear DNA damage repair. We performed a PCR-based DNA damage assay to assess the role of Uve1 in mitochondrial and nuclear DNA repair post-UV stress. The assay is based on the principle that damaged DNA impedes the progression of Taq polymerase on the template DNA in a PCR reaction [42]. Hence, there is an inverse relationship between the amount of DNA damage and PCR amplification products. Long template size increases the sensitivity of assay, as the longer the DNA the more chances of encountering damage (dimers). Small template amplification serves as a control, minimizing chances of encountering a damaged DNA strand, and is used for normalization of the amount of starting DNA or mitochondrial DNA copy number.

**Figure 7:**
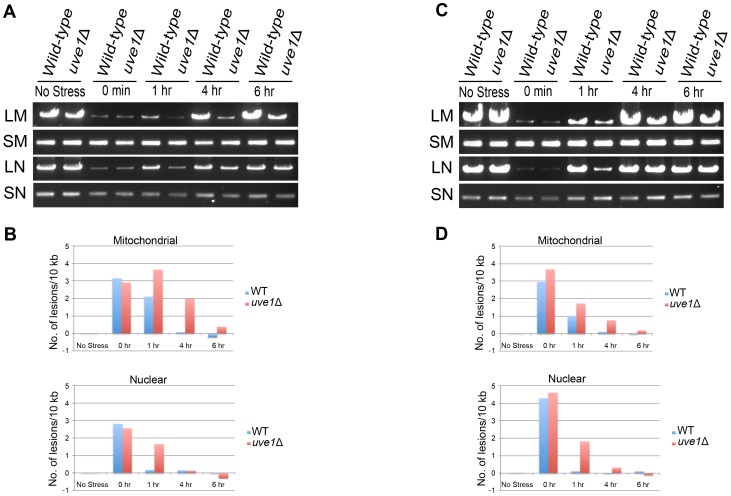
Uve1 is required for efficient repair of mitochondrial DNA damage post UV stress. Two independent experiments were carried out on different days. (A, C) Agarose gels for the PCR amplification of mitochondrial and nuclear DNA on template DNA from undamaged control cells and template DNA from cells exposed to UV stress 50 J/m^2^. *C. neoformans* var. *grubii* strains KN99α (WT) and AI191 (*uve1*Δ) PCR amplification pattern for no stress, 0 hour, 1 hour, 4 hour and 6 hour post UV stress recovery. DNA amplification of long mitochondrial (LM) DNA, short mitochondrial (SM) DNA, long nuclear (LN) DNA and short nuclear (SN) DNA. (B, D) Graphical representations of DNA damage experiments in panels A and C, respectively. X-axis represents number of lesions/10 kb and Y-axis represents time in hours.

We compared DNA damage between the nuclear genome and mitochondrial genome of UV treated samples for both wild type and *uve1*Δ (Figure 7). After one hour there is delayed repair for both nuclear and mitochondrial genomes in the *uve1*Δ strain compared to wild type, probably reflecting retrograde signaling between the mitochondria and nucleus or that repair pathways of the nuclear genome can be ATP dependent [43]–[48]. The key observation is that in the *uve1*Δ strain there is lag in the mitochondrial repair. For later time points of 4 hour and 6 hours, the *uve1*Δ strain repair of mitochondrial genome is delayed as compared to wild type strain, which returned to normal (Figure 7). At 6 hours recovery, amplification of the mitochondrial genome in the wild type is as it was prior to DNA damage, but for *uve1*Δ the lesion damage still persists. However, the *uve1*Δ strain shows more efficient repair of lesions in the nuclear genome. For the 4 hour and the 6 hour time points, as the repair process of the mitochondrial genome initiates in *uve1*Δ by some unknown mitochondrial DNA repair enzymes, the repair of the nuclear genome is faster and comparable to wild type levels. These data implicate Uve1 in the efficient repair of UV-damaged mitochondrial DNA, with evidence for a complex interplay between mitochondrial and nuclear repair and the contributions of other repair pathways.

### Overexpression of Uve1 rescues the UV sensitive phenotype of *bwc1* mutants

**Figure 8:**
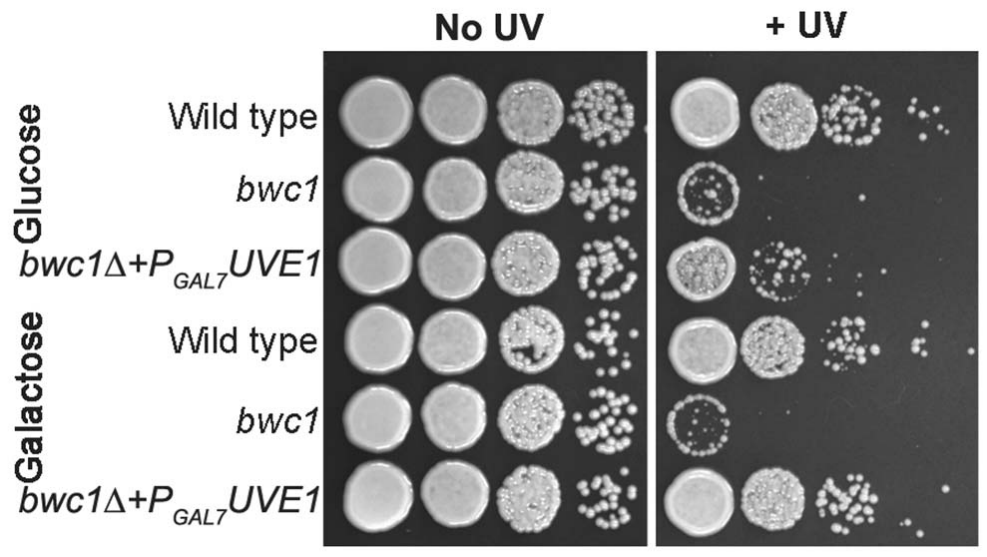
*UVE1* overexpression rescues the UV sensitive phenotype of *bwc1*Δ mutants in *C. neoformans*. Ten-fold serial dilutions for different strains of *C. neoformans* var. *neoformans* grown at 30°C for 2 days. Left panel untreated control and right panel treated with UV dose of 120 J/m^2^. Top three strains (JEC21, AI5 and AISVCN53) grown overnight in 2% glucose and bottom three strains (JEC21, AI5 and AISVCN53) grown overnight in 2% galactose before inoculating onto YPD plates.

No induction of the *UVE1* transcript encoding the functional form of the protein was observed under light conditions in *bwc1*Δ mutants (Figure 2). We examined if *UVE1* is a direct target of the Bwc1-Bwc2 complex through two approaches. First, we tested if overexpression of Uve1 could rescue the UV hypersensitive phenotype of the *bwc1*Δ mutant in *C. neoformans*. Uve1 from var. *neoformans* was expressed under a galactose-inducible promoter (P*_GAL7_*) in the *bwc1* deletion mutants of var. *neoformans* and var. *grubii*. The strains were grown overnight in media with glucose or galactose as the primary carbon source, serial diluted and plated, and UV sensitivity tests performed. Results from the var. *neoformans* strains are illustrated in Figure 8, and var. *grubii* in Figure S7. Uve1 overexpression rescued the UV sensitive phenotype of *bwc1*Δ as comparable to wild type when induced by galactose. In contrast, there was only slight rescue in strains grown in non-inducing glucose (Figure 8). For the *bwc1*Δ+P*_GAL7_-UVE1* overexpression strains and wild type strains we performed northern analysis to check the levels of *UVE1* induction (Figure S8). The levels of *UVE1* transcripts were comparable between the galactose-induced P*_GAL7_*-*UVE1* and the light-induced wild type strains. These results provide one piece of evidence for the Bwc1-Bwc2 complex directly controlling UV resistance in *C. neoformans* through regulation of the effector protein Uve1.

### Recombinant Bwc2 binds to the promoter of *UVE1* of *C. neoformans*

**Figure 9:**
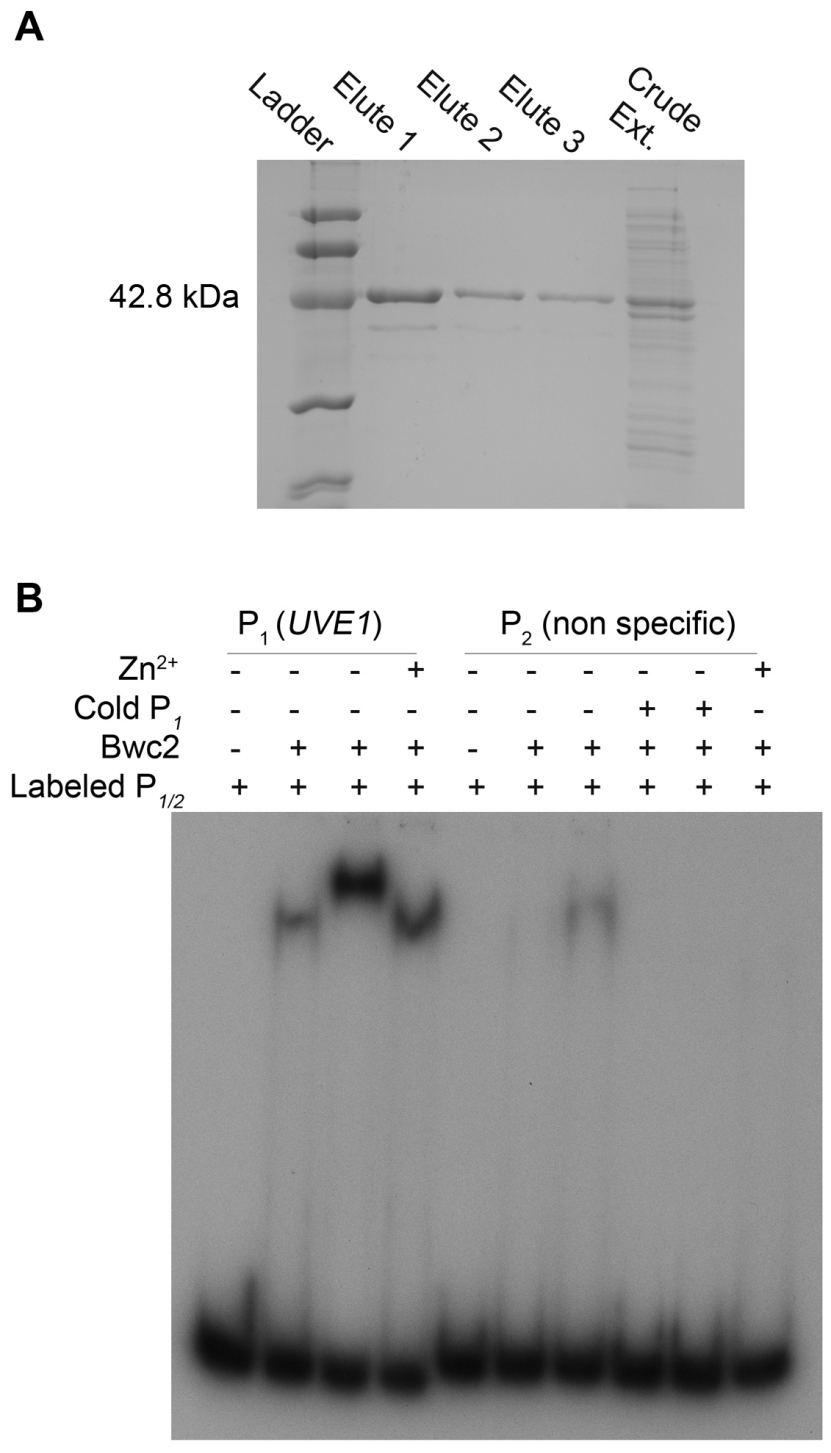
Bwc2 binds to the *UVE1* promoter. (A) SDS-PAGE gel for *C. neoformans* recombinant (His)_6_-Bwc2. Left to right, lane 1 is the low molecular weight Bio-Rad protein ladder, lane 2, 3, 4 are purified recombinant Bwc2 eluted fractions. Lane 5 is crude unpurified protein extract. (B) Gel mobility shift assay for a fragment of the JEC21 *UVE1* promoter with purified Bwc2. Left to right, lane 1 contains just the *UVE1* promoter P_1_ (P*_UVE1_*), lane 2, 3 and 4 contains P_1_+Bwc2 20 µg, 50 µg and 20 µg+Zn^2+^ respectively. Lane 5 is loaded with the non-specific radiolabeled probe P_2_. Lane 6, 7 are P_1_+Bwc2 20 µg and 50 µg. Lane 8, 9 are competition with the increasing amount of specific probe cold P_1_ to negate any binding observed in lane 7. Lane 10 is Bwc2 and P_2_ with Zn^2+^.

The second piece of evidence that *UVE1* is a direct target of Bwc1-Bwc2 comes from gel mobility shift assays. Bwc2 has a C-terminal GATA-type zinc finger whose binding target sites are not known in *C. neoformans*. We searched the promoter of *UVE1* for putative Bwc2 binding sites based on those used by the WCC of *N. crassa* [11], [14], [49]. For instance, one light regulated element (LRE) found in the *frq* promoter is TCGATC CGCTCGATCCCCT , with the underlined nucleotides similar to a TCGATC TTCATCTCGATCTCCA sequence found in the promoter of *C. neoformans UVE1*. We amplified and radiolabeled the *UVE1* promoter region with this site and performed gel mobility shift assays with recombinant Bwc2 (amino acids 26 to 383) expressed and purified from *Escherichia coli* (Figure 9A). A retardation in gel migration of the *UVE1* promoter DNA was observed, that increased with higher Bwc2 concentration or by adding zinc which is expected for a zinc finger protein (Figure 9B). The nature of the higher mobility forms is unknown, but may represent aggregation of Bwc2 monomers. Control interactions using a non-specific DNA fragment confirmed the specificity of Bwc2 for the *UVE1* promoter. These observations indicate that the *UVE1* promoter is a direct target for Bwc2 binding.

## Discussion

Light influences diverse aspects of fungal biology, presumably by acting on unique pathways in specific species. However, the potential for conserved regulation also exists, and this is predicted to reflect the original selective pressure(s) and current maintenance of light-sensing in extant fungi. Some responses to light in ascomycete fungi relate to protection against damage caused by light. For instance, expression of the DNA repair enzyme photolyase and genes for biosynthesis of carotenoid pigments are often induced by light. There are previous reports of links between light via its input in circadian rhythms with DNA repair in fungi, as well as in mice and humans. For instance PRD-4 is a checkpoint kinase 2 homolog in *N. crassa* that is regulated by the WCC and contributes to DNA repair [21]. Another DNA repair protein, XPA, has been shown to have circadian rhythm dependent oscillations in mouse brain [50], [51]. However, between 2.8–6% of genes are regulated at the transcript level in response to a light exposure in ascomycete species [11], [52], [53], yielding a long list of candidate genes for further analysis. In contrast, the basidiomycete *C. neoformans* may serve as a simpler model for understanding the evolution of light-sensing in fungi, because (a) it does not encode a photolyase gene and pigmentation is not induced by light, (b) few genes are induced in response to light at the transcript level [19], (c) there is no evidence of photoadaptation [17], a trait that influences the intensity of the response to light, and (d) the White collar complex contains only one protein with a zinc finger DNA binding domain [17], [18].

**Figure 10:**
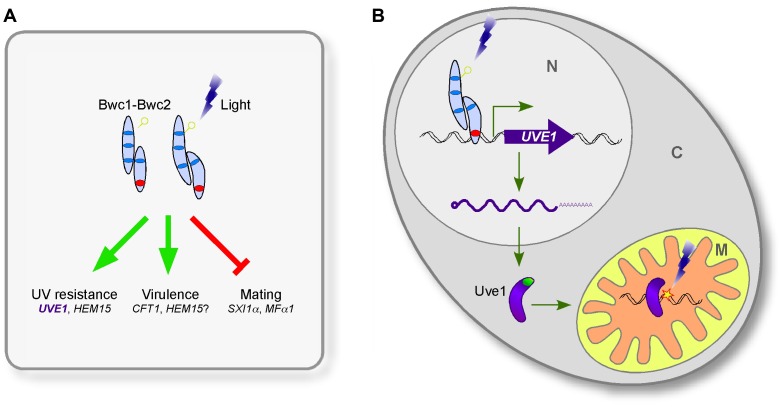
The involvement of Uve1 in the photosensory response of *C. neoformans*. (A) Bwc1-Bwc2 influences three aspects of *C. neoformans* biology: mating, UV tolerance, and virulence. Uve1 impacts one of these three traits. (B) Uve1 endonuclease protects *C. neoformans* under UV radiation stress by protecting its mitochondrial genome. Photoreceptor Bwc1 senses blue/UV light, undergoes change in its flavin-binding domain, is activated, and forms a complex with Bwc2, a zinc finger transcription factor. Bwc2 with Bwc1 binds the *UVE1* promoter to activate its transcription. Uve1 protein has a mitochondrial localization signal (green), and is transported to mitochondria. In mitochondria, on sensing the UV-induced DNA damage (star) Uve1 binds to damaged DNA and initiates repair by the UVDE DNA damage repair pathway. N nucleus; C cytoplasm; M mitochondria.

Here, we identify the *UVE1* gene as a downstream target of the WCC in *C. neoformans*, and show that homologs are also light regulated in species that represent two other major branches in the fungal kingdom. We suggest that *UVE1* acts as the key factor controlling the UV sensitive phenotype caused by mutating *BWC1* or *BWC2* in *C. neoformans* (Figure 1, Figure 10, Figure S4), and likely plays similar roles in other fungi to survive the deleterious effects of sunlight, which has UV wavelengths as an inevitable DNA damaging component.

Northern blot analysis and characterization of 5′ and 3′ ends of *C. neoformans* var. *neoformans UVE1* showed two transcripts of differing size for the *UVE1* gene. The functional complementation experiments (Figure 6) in *S. pombe uve1*Δ by the homologous *UVE1* long isoform implies that the *C. neoformans* protein has similar DNA repair activities; such as against cyclobutane pyrimidine dimers, 6-4 photoproducts, apurinic/apyrimidinic sites, and stretches of single-stranded DNA nicks or gaps. Moreover the localization of *C. neoformans* Uve1-GFP in *S. pombe* is in part nuclear rather than mitochondrial (Figure 6). This explains the functional complementation of the UV stress tolerance phenotype in *S. pombe uve1*Δ strains, as in *S. pombe* the nuclear UVER pathway is attributed for survival under UV stress [32]. The *UVE1* short isoform did not show any functional complementation. Bioinformatic analysis to identify the active domain of *UVE1* (pfam03851) provides a possible explanation for the inactivity of the short isoform, because it does not encode the complete conserved region (Figure 3, Figure S3). Another possibility may be the absence of subcellular localization signals on the dark form, such that the protein is rendered inactive due to improper compartmentalization. Correct subcellular localization of proteins involved in DNA repair has been implicated in countering genotoxic stress [54], as improper localization can result in loss-of-function and may even lead to disease development in humans [55], [56].

The light isoform of Uve1 in *Cryptococcus* localizes to the mitochondria, as shown by Uve1-GFP fusion studies. *C. neoformans* is an obligate aerobe: it cannot survive loss of mitochondrial function from something like unrepaired DNA damage. Based on the following evidence: (a) no observed localization of Uve1 in nucleus (Figure S6); (b) localization of Uve1 to mitochondria (Figure 5); (c) strains with the *UVE1* gene deleted exhibited reduced survival under UV stress and Uve1-GFP partially complements the UV sensitive phenotype of the *uve1*Δ strain; and (d) reduced mitochondrial DNA damage repair in *uve1*Δ strain comparatively to the wild type strain (Figure 7) suggest that Uve1 in *Cryptococcus* is required for protection of mitochondrial DNA for survival under UV stress. Overexpression of *UVE1* in *bwc1* mutants of either var. *grubii* or var. *neoformans* restores UV sensitivity to the wild type level (Figure 8), and recombinant Bwc2 physically binds to the promoter of *UVE1* to cause a gel mobility shift (Figure 9B). These data further corroborate the hypothesis that *UVE1* is a direct downstream target of Bwc1.

The light-induced genes in *C. neoformans* were previously sought by a whole genome microarray expression analysis in var. *neoformans* [19]. The *UVE1* gene was not identified in that study although *UVE1* transcript data are present for five of the six biological replicates, with an average 1.15 fold difference between dark and light treatment. By contrast, quantification by ImageJ of our northern blot data normalized to actin levels indicates that the longer isoform is upregulated about 16 fold in the light (Dataset S1). *UVE1* remained undetected in microarray experiments because the 70-mer probe on the array is common to both light and dark transcript isoforms (Figure 3). The dark isoform must be under control of another transcription factor, using elements within the light isoform to drive transcription. These findings demonstrate one limitation of the microarray technique in comparison to more comprehensive transcript analysis techniques like tiling arrays or RNA-seq, or to conventional hybridization techniques like northern blotting that can detect alternative transcripts.

Two other phenotypes associated with mutation of *BWC1* or *BWC2* in *C. neoformans* are loss of the inhibition of mating by light and reduced virulence. To further verify the role of Uve1 in other *BWC* related phenotypes, we examined the role of Uve1 in *C. neoformans* mating by crossing *uve1*Δ strains of both mating types under light and dark conditions. We did not find any contribution of this gene in mating, as the *uve1*Δ strains behaved like wild type for repression of mating by light (Figure S2). Similarly, the phenotype of the *uve1*Δ mutant in animal studies does not phenocopy that of *bwc1*Δ or *bwc2*Δ. A large-scale analysis of virulence has been undertaken in *C. neoformans*, measuring competitive survival of strains in mouse lungs [38]. Both *bwc1*Δ and *bwc2*Δ strains showed reduced proliferation, consistent with their reported role in virulence [17]. In contrast, the *uve1*Δ mutant had no defect in this virulence assay. We corroborated these results in a wax moth larvae model of virulence (Figure S2). We also examined a possible role of *UVE1* under oxidative stress based on the mild phenotype observed in *S. pombe* [31], but did not find any phenotypic difference in the *uve1*Δ strains compared to wild type. Thus, we postulate that Bwc1-Bwc2 modulates its function via more than one downstream target (Figure 10A); of which those for virulence and mating remain to be discovered in future studies. We propose a model for the relationship between light-sensing via the WCC and Uve1 function in the mitochondria in *C. neoformans* (Figure 10B). It is possible that this model applies also to other fungal species for the protection of mitochondrial, nuclear or both genomes under UV stress.

The White Collar complex is conserved across the fungal kingdom, with homologs present in the chytrids, Mucoromycotina, Glomeromycotina, Ascomycota and Basidiomycota. However the complex has been lost in some fungal lineages, like its absence from the Saccharomycotina [1], [2], [57]. One important question is whether any WCC downstream targets are conserved. Transcript comparisons by northern blot analysis in *N. crassa* and *P. blakesleeanus*, members of the Ascomycota and Mucoromycotina, demonstrate that *UVE1* homologs are photo-regulated in at least one species in each of these fungal groups. The *UVE1* homolog is also induced by light, as measured by microarray studies, in *Aspergillus nidulans* and *N. crassa* [11], [53]. Absence of regulation in *bwc1* and *madA-madB* mutants in *N. crassa* and *P. blakesleeanus* further implicate White Collar-dependent regulation of *UVE1*. This suggests that in fungi that have White Collar, *UVE1* is regulated in a light-dependent manner, and this regulation is lost or alternative regulation evolved in fungal species missing White Collar proteins. The presence of LOV domain containing flavin-binding photoreceptor proteins and *UVE1* homologs in bacteria, like *Bacillus subtilis* [25], [58], warrant further examination if through convergent evolution the LOV domain type photoreceptors might be involved in regulation of *UVE1* expression even more widely.

The repair of photo-damage by Uve1 is conserved in many fungi and important under UV stress, irrespective of the presence of base excision (BER) or nucleotide excision repair (NER) pathways. Repair of DNA damage from UV by Uve1 is faster in comparison to NER [40]; under ancient environments in which ultraviolet levels were higher than today Uve1 could have provided a selective advantage. We estimate from genome sequencing projects that 95% of the fungal genomes encoding *WC-1* also encode a copy of *UVE1*. Many of the exceptions have homologs of photolyase present in their genome, which may play an equivalent role as Uve1, and can also repair mitochondrial DNA [59], [60].

In summary, light triggers a number of physiological and morphological changes in fungi. The advantages of using light as a signal that are conserved have remained unclear although there is increasing evidence for a role in protecting cells from damage. Here, we demonstrate that protection of DNA, including the mitochondrial genome, through photo-regulation of Uve1 provides a benefit that is present in fungi that are able to sense light through the White Collar Complex.

## Materials and Methods

### Generation of *UVE1* knockouts and their complementation in *C. neoformans*

Gene knockout cassettes were constructed by fusion of around 1000 bp flanks 5′ and 3′ of the *UVE1* gene with nourseothricin acetyltransferase (*NAT*) coding sequence for strain JEC21 (var. *neoformans*, serotype D) and neomycin phosphotransferase (*NEO*) coding sequence for strain KN99α (var. *grubii*, serotype A). Oligonucleotide primer sequences are listed in Table S1. To make JEC21 *uve1*Δ, 5′ and 3′ gene flanks were amplified by primer set AISV030/AISV034 and AISV032/AISV035, respectively, using JEC21 genomic DNA. To make KN99α *uve1*Δ, 5′ and 3′ gene flanks were amplified by primer set ai830/ai831 and ai832/ai833, respectively, using KN99α genomic DNA. The *NAT* and *NEO* ORFs were amplified by primer set ai290/ai006. Overlap PCR was performed to obtain 5′-*UVE1*-*NAT*-*UVE1*-3′ and 5′-*UVE1*-*NEO*-*UVE1*-3′ cassettes by mixing equimolar ratio of 5′-*UVE1*, *NAT*, *UVE1*-3′ for JEC21 and 5′-*UVE1*, *NEO*, *UVE1*-3′ for KN99α. Primers used to perform overlap PCR were AISV030/AISV032 and ai830/ai833 for JEC21 and KN99α, respectively. About 2 µg of 5′-*UVE1*-*NAT*-*UVE1*-3′ or 5′-*UVE1*-*NEO*-*UVE1*-3′ cassette was transformed into strains JEC21 and KN99α using biolistic delivery with a PDS 1000/He particle delivery system (Bio-Rad, Hercules, CA) [61]. Gene replacement was confirmed by PCR and Southern blots for the correct integration of the gene cassette. Strains and genotypes are provided in Table S2.

For complementation of *UVE1* in serotype A, a wild type copy of *UVE1* was amplified with primers ALID0001 and ALID0002 and cloned into the pCR2.1 TOPO plasmid. The insert was excised with BamHI-XhoI and subcloned into the BamHI-SalI site of pPZP-NATcc. The plasmid was transformed into strain AI191 (*uve1::NEO*) by biolistics, with positive transformants selected for growth on yeast extract-peptone-dextrose (YPD)+nourseothricin (100 µg/ml) plates. The plasmids used or constructed in this study are listed in Table S3.

The UV sensitivities of strains were tested by applying UV stress in an XL-1500 UV cross linker (Spectronics Corporation, Lincoln, NE). Unless otherwise stated, strains were exposed to the laboratory ambient light (400–800 LUX) during experiments.

### Northern blot analysis of transcript levels of *UVE1* homologs

Cultures were prepared for *C. neoformans* strains KN99α, AI81 (*bwc1*Δ), JEC21, AI5 (*bwc1*Δ), *C. gattii* (R265), *S. pombe* L972, *N. crassa* wild-type FGSC 4200, *N. crassa* (*wc-1*) FGSC 4398, *P. blakesleeanus* wild-type NRRL1555, and *P. blakesleeanus* (*madA madB*) mutant L51. All strains of each species were plated with equal optical density or numbers of spores in duplicates on 15 cm diameter petri dishes containing YPD, and kept in darkness. For *N. crassa* only, 50 ml liquid cultures were grown in 50 ml YPD medium. Cultures were grown for 23 h or 47 h depending on the growth kinetics of the species. On completion of 23 h or 47 h, one of the sets was exposed to cool white light (dual Sylvania 4100 K 32W bulbs) of 1600–2600 Lux for 1 h and the other set left in the dark. On completion of the 24 h or 48 h time periods both the light and dark cultures were scraped in the light or under safe red light (GBX LED safelight, Kodak, Rochester, NY). *N. crassa* cultures were harvested directly from the liquid medium. All cultures were pelleted, frozen using dry ice+ethanol, lyophilized and stored at −80°C.

Total RNA was isolated using Trizol reagent (Invitrogen, Grand Island, NY). To address the low transcript abundance of *UVE1*, total RNA was further purified for polyA mRNA isolation starting with 1 mg total RNA, using the PolyATract Kit (Promega, Madison, WI), except for *P. blakesleeanus* and *C. gattii* where 40 µg of total RNA were used. RNA samples were resolved on 1.4% agarose denaturing formaldehyde gels and blotted on to Zeta Probe membrane (Bio-Rad). Probes for northern analysis were amplified using specific primer sets (Table S4) and radiolabeled with [α-^32^P] dCTP (PerkinElmer, Waltham, MA) using the RediPrime II labeling kit (Amersham, Pittsburg, PA). The blots were stripped and re-probed with fragments of actin homologs as loading controls. Autoradiograms were scanned, and transcript levels were compared by ImageJ analysis (Dataset S1).

### Subcellular localization of Uve1

The localization of the two isoforms of Uve1 within the cell was assessed by fusions to green fluorescent protein (GFP). *C. n.* var. *neoformans* strain JEC21 genomic DNA was used as the template to amplify the *UVE1* light (L) isoform using primers AISV001/AISV003 and *UVE1* dark (D) isoforms using primers AISV002/AISV003. The histone 3 promoter for *Cryptococcus* (P*_H3_*) and *GFP-NAT* were amplified from the pPZP-GFP-NATcc plasmid using ai255/AISV005 (overlap primer for L) or ai255/AISV006 (overlap primer for D) for P*_H3_*, and AISV004/ai256 for *GFP-NAT*. The overlap construct was amplified by mixing equimolar ratios of the three amplicons for the light and dark isoforms using primers M13F and M13R. About 2 µg of gel purified *UVE1* L and D overlap constructs were transformed into strain AI191 by biolistics. Positives clones were selected by their growth on YPD+100 µg/ml nourseothricin plates and confirmed by PCR, DNA sequencing, western blotting for GFP, and fluorescence signal.

Strains AISVCN28 and AISVCN02 were used for localization of the Uve1 light and dark isoforms fused to GFP. Strains were stained with MitoTracker Red CMXRos (Invitrogen) at 3 nM, kept in the dark for 20 min, washed and suspended in phosphate buffered saline (PBS) and used for microscopy. Cells were imaged using Olympus confocal microscopes FLUOVIEW FV10i or FV300.

### DNA damage assay

Cultures for *C. neoformans* strains KN99α (wild type) and AI191 (*uve1*Δ) were grown overnight in YPD and washed with distilled water. Cells were suspended in phosphate buffered saline to 4×10^4^ cells/ml. For each strain totals of 150 ml cells were distributed in 30 ml aliquots for time points 0 min (after stress), 1 h, 4 h, 6 h and control (no stress). For each aliquot the cells were placed in 15 cm petri dishes and exposed to UV light (50 J/m^2^) using a UV cross linker. Immediately after the UV stress, cells were transferred to 50 ml tubes and kept on ice. Control and 0 min cells were pelleted and frozen in liquid nitrogen. For 1 h, 4 h and 6 h time points, cells were re-suspended in YPD and incubated at 30°C for these respective times, then centrifuged to pellet and snap frozen. All samples were lyophilized, and DNA was extracted by the CTAB buffer method [62].

The relative DNA damage to the mitochondrial and nuclear genomes were assessed using a PCR assay based on established methods [42]. Concentrations of DNA samples from each treatment were standardized by measuring them by spectrophotometry and making appropriate dilutions. Primers used for amplification of fragments of the mitochondrial genome were AISV87/AISV91 (11 Kb). PCR conditions for long mitochondrial PCR were 94°C 4 min, 23 cycles for 98°C 10 s, 68°C 15 min, and a final extension of 72°C 10 min using Ex Taq (Takara, Kyoto, Japan). For nuclear long amplification (8 kb) primers were AISV85/AISV95. Conditions for long nuclear PCR were 94°C 4 min, 23 cycles for 94°C 20 s, 58°C 20 s, 72°C 6 min and a final extension of 72°C 7 min. Short amplification primers for mitochondrial genome were AISV89/AISV99 and for the nuclear genome AISV85/AISV97 amplifying about 250 bp. PCR conditions for small mitochondrial and nuclear amplicons were 94°C 4 min, 23 cycles for 94°C 20 s, 55°C 20 s, 72°C 1 min, and a 72°C 7 min final extension. All PCR amplicons were resolved on agarose gels, and intensities were quantified using ImageJ software. DNA damage was compared by calculating relative amplification of large PCR fragments of the UV treated samples to that of the respective untreated controls using the method reported in reference [42], and adjusting for differences between nuclear and mitochondrial amplicon sizes.

### Virulence assay in wax moth larvae

The *Galleria mellonella* virulence assay followed methods that were previously described [63]. Overnight cultures in YPD medium for strains KN99α, AI191 and AI181 were washed three times with PBS. Cells were suspended in PBS to 2×10^7^ cells/ml. For each strain 11–12 larvae were injected with 5 µl of the cells, as well as the control PBS. Wax moth were incubated at 37°C and survival monitored daily.

### Complementation tests with *C. neoformans UVE1* isoforms in a *S. pombe uve1* knockout strain

An *S. pombe uve1* knockout strain was constructed to serve for the functional analysis of *UVE1* isoforms from *C. neoformans*. For the construction of the gene knockout cassette, genomic DNA from *S. pombe* strain L972 was used to amplify around 320 bp 5′-*uve1* and 300 bp 3′-*uve1* fragments using primer pairs AISV007a/AISV009 and AISV010/AISV011, respectively. The KanMX fragment was amplified using primer set AISV007/AISV008 from plasmid pFA6a-GFP(S65T)-kanMX6. Overlap PCR was performed to generate the gene knockout cassette using primer set AISV007a/AISV011. Around 2 µg of the PCR construct were transformed into *S. pombe* (strain MM72-4A *ura4-D18 h^−^*) by lithium acetate transformation and cells plated on to YPD+100 µg/ml G-418. Gene knockouts were confirmed by PCR and Southern blotting. *S. pombe uve1* knockout strain AISVSP1 was selected for *C. neoformans UVE1* complementation studies.

*UVE1* cDNA was reverse transcribed from RNA of *C. neoformans* strain JEC20 using Superscript III First strand Synthesis System (Invitrogen), as per company instructions (JEC20 is isogenic to JEC21, with a different *MAT* allele; [64]). The synthesized cDNA was amplified by site directed mutagenesis to abolish an NdeI site inconvenient for subcloning while conserving the encoded amino acid residue, and to introduce NdeI and BamHI restriction sites at the start and end of the *UVE1* gene. Primers used for amplification of fragment 1 of the L and D form were AISV014/AISV013 and AISV015/AISV013, respectively. Primers used for amplification of fragment 2 were AISV012/AISV016. Overlap PCR was performed to amplify full L and D genes from fragment 1 and 2, using primers AISV014/AISV016 for *UVE1* L form and AISV015/AISV016 for *UVE1* D form. The NdeI and BamHI digested cassettes were ligated into the NdeI-BamHI site in the pREP42 vector enabling expression from an *nmt* promoter [39]. Positive clones were confirmed by sequencing. Plasmids containing *UVE1* L and D isoforms were transformed into *S. pombe* strain AISVSP1 (*ura4-D18 uve1::kanMX*) by the lithium acetate method. Empty vector pREP42 was transformed into strain AISVP1 as a control. Positive *S. pombe* transformants were selected on minimal medium without uracil and were confirmed by PCR.

### *C. neoformans* Uve1-GFP localization in *S. pombe*

*C. neoformans* var. *neoformans* Uve1 (L) C- terminal GFP localization in *S. pombe* was done by fusion of *UVE1* (L) to GFP by overlap PCR. For fragment 1, *UVE1* (L) was amplified from pREP42-*UVE1* (L) using primers AISV014/AISV003 and the fragment 2, GFP, was derived from pPZP-GFP-NATcc using primers AISV004/AISV066. Overlap PCR joining fragments 1 and 2 was performed by primer set AISV014/AISV066. The overlap PCR product was cloned into pCR 2.1 TOPO, and transformed by heat shock into *E. coli* DH5α. Positive clones were selected and sequenced. A plasmid containing the desired Nde1-*UVE1*-GFP-BamHI overlap was digested with NdeI and BamHI. The Nde1-*UVE1*-GFP-BamHI digest was ligated with NdeI and BamHI digested pTN157. Plasmid pTN157-*UVE1*-GFP was transformed into *S. pombe* strain AISVSP1 (genotype *ura4-D18 uve1::kanMX*) by the lithium acetate method. Positive *S. pombe* transformants were selected on minimal medium without uracil and were confirmed by PCR. Transformants were examined for fluorescence signal and their UV resistant phenotype. Confocal microscopy was performed for strain AISVSP15. For both mitochondrial and nuclear staining, cells were grown in Edinburgh Minimal Medium (EMM). Mitochondrial staining was performed with MitoTracker Red CMXRos (Invitrogen) at a final concentration of 3 nM in water, kept in the dark for 20 min, washed and suspended in PBS and used for microscopy. Hoechst 33342 was used to stain the nucleus. Cells grown in EMM were washed and suspended in Hoechst (1 µg/ml in water) for 10 min. Cells were washed and suspended in PBS, and microscopy was performed.

### UV survival experiments for *S. pombe*

Overnight cultures for strains L972, AISVSP1, AISVSP2, AISVSP3, AISVSP4 and AISVSP15 in YES media were subcultured the following day. Strains in exponential phase were dotted on YES medium in ten-fold serial dilutions. One set of plates was exposed to UV light (120 J/m^2^) using the UV cross linker, and another plate kept unexposed. Both UV treated and unexposed sets were incubated at 30°C for 2 days. For UV dose response experiments exponentially growing cells for strains at the same optical density were ten-fold serially diluted and equal volumes for all dilutions of the cells were plated on YES media. The control was kept unexposed to UV and others were exposed at UV doses of 60, 120 and 180 J/m^2^. All plates were incubated at 30°C for 3 days, and colony forming unit data analyzed for percentage survival.

### *UVE1* over-expression in *C. neoformans bwc1* mutants

The *UVE1* gene was over-expressed in *bwc1* knockout backgrounds to assess if *UVE1* can rescue the UV sensitive phenotype of *bwc1* mutation. A fusion cassette of the JEC21 *GAL7* promoter (P*_GAL7_*) and *UVE1* gene was made by overlap PCR. The *GAL7* promoter was amplified by primer set AISV025/AISV028 and *UVE1* was amplified using primer set AISV027/AISV026 on JEC21 genomic DNA. The P*_GAL7_*-*UVE1* fusion cassette was amplified by mixing *GAL7* promoter and *UVE1* PCRs in equimolar ratios by primer set AISV025/AISV026. The P*_GAL7_*-*UVE1* fusion cassette was cloned in a TA vector (pCR 2.1 TOPO; Invitrogen), and transformed by heat shock in *E. coli* Top10 cells. Positive clones were selected and sequenced. A plasmid containing P*_GAL7_* -*UVE1* was digested with BamHI and XhoI. The fragment was ligated with pPZP-NEO *Agrobacterium* vector digested with the same enzymes, and transformed into Top10 cells. Positive clones were selected by their growth on kanamycin and verified by PCR and restriction digestion. The pPZP-NEO-P*_GAL7_*-*UVE1* vector was transformed by electroporation into *A. tumefaciens* strain EHA105. Selected positive *Agrobacterium* transformants were co-cultured with *C. neoformans* strains AI5 and AI81. Positive transformants were selected for growth on YPD+G-418+cefotaxime plates. Transformants were cultured overnight in yeast nitrogen base (YNB) medium +2% galactose or 2% glucose, and 10-fold serial dilutions placed on YPD medium plates. Dotting was performed in duplicate and one set was exposed to UV radiation at 120 J/m^2^. Both sets were incubated at 30°C for 2 days.

### Expression of recombinant Bwc2 and electrophoretic mobility shift assays

JEC21 RNA was reverse transcribed to make cDNA using the Superscript III First strand Synthesis System (Invitrogen). A fragment of *BWC2* cDNA was amplified using primers AISV040/AISV041, containing BamHI and EcoRI restriction sites. The amplicon was digested with BamHI and EcoRI, and ligated into the pRSETA vector (Invitrogen) digested with the same enzymes. Top10 cells were transformed with the ligation product. An error free clone was identified by sequencing. The plasmid containing *BWC2* was transformed into BL21(DE3)pLysS cells and selected on ampicillin+chloramphenicol SOB medium plates. Protein induction and expression using 1 mM IPTG was performed as described for the pRSET expression system by Invitrogen. The (Histidine)_6_-tagged Bwc2 protein was semi-purified using Pure Proteome Nickel Magnetic beads (Millipore Corporation, Billerica, MA) as per the manufacturer's instructions.

For electrophoretic mobility shift assays (EMSA), a 244 bp (P_1_) region 5′ of the start codon of *UVE1* containing putative Bwc2 binding sites was amplified using primer set AISV019/AISV020. A control nonspecific DNA fragment (NS) of 278 bp was amplified from pRS426 vector using primers ALID1229/ALID1230. About 600 ng of the amplified fragment from *UVE1* promoter region and nonspecific probe was radiolabeled with [γ-^32^P] dATP (PerkinElmer) using T4 polynucleotide kinase (New England Biolabs, Ipswich, MA). Labeling conditions were 1X T4 polynucleotide buffer, 5 µl 6000 Ci/mmol γ-^32^P ATP, 10 units T4 polynucleotide kinase in a 50 µl reaction mixture at 37°C for 30 min. The radiolabeled probes were purified using PCR purification columns (Qiagen, Germantown, MD). The composition of 5X EMSA binding buffer used was 20% w/v glycerol, 5 mM MgCl_2_, 250 mM NaCl, 2.5 mM EDTA pH 8, 10 mM DTT with or without 12 µM ZnSO_4_ (reference [65] with slight modifications). 20 to 50 µg of purified Bwc2 protein were pre-incubated with 1X EMSA binding buffer for 20 min at room temperature for all reactions. Total reaction mixture of 20 µl consisted of 1X EMSA binding buffer, 20 to 50 µg of purified protein, cold probe (if added), 1 µl of radiolabeled probe and 1X phosphate buffer, followed by 50 min incubation at room temperature. For competition reactions, 600 ng and 900 ng of the cold probes were included in the reaction mixture prior to addition of radiolabeled probes. At the end of 50 min incubation, samples were transferred to ice followed by addition of EMSA loading dye. Samples were loaded on 6% polyacrylamide Tris-borate-EDTA (TBE) gels (Invitrogen), and run at 130 V for 2 h in 1X TBE at 4°C. Autoradiography was performed by exposure to Gene Mate Blue ultra autoradiography films.

## Supporting Information

Dataset S1 ImageJ quantification for northern blots. Autoradiographs were scanned, pixel intensity measured for the *UVE1* homologs or actin loading controls, and relative expression levels provided for comparison between dark and light grown cultures.
(XLSX)

Figure S1 Different DNA damage chemicals, UV and heavy metal stress for the WT, *uve1*Δ and *uve1*Δ+*UVE1 C. n.* var. *grubii* strains. The order of the strains from top to bottom for each panel is KN99α (WT), AI191 (*uve1*Δ) and AI198 (*uve1*Δ+*UVE1*). Ten-fold serial dilutions for strains grown on YPD media at 30°C for 2 days. The stresses are UV (120 J/m^2^), tBOOH t-butyl hydroperoxide (0.6 mM),H_2_O_2_ hydrogen peroxide (1 mM), MMS methyl methanesulfonate (0.025%), Paraquat (0.5 mM), and cobalt chloride (60 µM).
(TIF)

Figure S2 The *uve1*Δ strain is unaffected in mating and virulence. (A, B) Crosses were set for *C. n.* var. *neoformans* and *C. n.* var. *grubii* strains. Yeast cells of opposite mating type were mixed on V8 medium and Murashige-Skoog medium, in light grown and dark grown condition for 3 to 4 days and photographed. (A) Mating for *C. n.* var. *neoformans*, top panel JEC21 (WT, *MAT*α) X JEC20 (WT, *MAT* **a**), middle panel AISVCN101 (*uve1*Δ, *MAT*α) X AISVCN104 (*uve1*Δ, *MAT* **a**) and bottom panel AI5 (*bwc1*Δ, *MAT*α) X AI6 (*bwc1*Δ, *MAT* **a**). (B) Mating for *C. n.* var. *grubii*, top panel KN99α X KN99**a**, middle panel AI191 (*uve1*Δ, *MAT*α) X AISVCN52 (*uve1*Δ, *MAT* **a**), and bottom panel AI81 (*bwc1*Δ, *MAT*α) X AI89 (*bwc1*Δ, *MAT* **a**). (C) Graphical representation for the percentage survival for wax moth larvae injected with the either PBS or 2×10^7^ cells/ml of KN99α, AI191 or AI81.
(TIF)

Figure S3 Alignment of Uve1 homologs from fungi, bacterium *Thermus thermophilus* and Archaea *Sulfolobus acidocaldarius*. Fungal species are *C. neoformans* var. *neoformans* (*C. n. neof.*), *C. neoformans* var. *grubii* (*C. n. grubii*), *S. pombe*, *N. crassa* and *P. blakesleeanus*. Mitochondrial localization sequences predicted by MItoProt are in green. Residues conserved in all species are marked by an asterisk. The two residues associated with the catalytic active site are highlighted in red. The start of the dark short isoform from var. *neoformans* is indictaed by the blue arrow.
(TIF)

Figure S4 Pre-exposure to light increases resistance to UV irradiation. Ten-fold serial dilutions for strains of *C. neoformans* var. *neoformans* grown at 30°C for 2 days after UV stress. Strains used are JEC21 (WT), AI5 (*bwc1*Δ) and AI51 (*bwc1*Δ+*BWC1*). Top three panels are strains grown in dark and given 2 h of white light (4,400 LUX) prior to UV stress. Bottom three panels are for the strains kept in constant darkness before UV stress.
(TIF)

Figure S5 UV sensitivity of DNA repair genes and their regulation by light. (A) Ten-fold serial dilutions for *C. neoformans* var. *grubii* strains, untreated or stressed with UV and grown at 30°C for 2 days. Order of *C. neoformans* var. *grubii* strains from top to bottom is KN99α, D320 (*rad27*Δ), D893 (*rad17*Δ), D1445 (*rad50*Δ), D287 (*msh201*Δ), D397 (*rad4*Δ), D1053 (*rad53*Δ), D759 (*uve1*Δ), D288 (*rad23*Δ), D594 (*mre11*Δ), D1344 (*rad10*Δ), D203 (*rad201*Δ), D293 (*rad6*Δ), AI219 (*rad1*Δ). (B) Northern blots for genes implicated in repair of UV damage, in *C. neoformans* var. *neoformans* and *C. neoformans* var. *grubii*. L and D represent light grown and dark grown conditions for the strains.
(TIF)

Figure S6 Subcellular localization of Uve1 (D)-GFP and Uve1 (L)-GFP from *C. neoformans* var. *neoformans* in *C. neoformans* var. *grubii*. (A) Uve1 (D)-GFP localization compared with MitoTracker, and (B) Uve1 (L)-GFP localization compared with nuclear Hoechst staining. The scale bar is 5 µm.
(TIF)

Figure S7 *UVE1* overexpression rescues the UV sensitive phenotype of *bwc1*Δ mutants in *C. n.* var. *grubii*. Three strains, KN99α (WT), AI81 (*bwc1*Δ) and AISVCN66 (*bwc1*Δ+P*_GAL7_*-*UVE1*), were grown overnight in YNB+glucose or YNB+galactose before inoculating onto YPD plates, and grown at 30°C for 2 days.
(TIF)

Figure S8 Northern blot and quantification of P*_GAL7_-UVE1* expression in *bwc1*Δ compared to the level in light-induced wild type. (A) Northern blot of *C. n.* var. *neoformans* from left to right, JEC21 (WT) grown on glucose, AI5 (*bwc1*Δ) grown on glucose, AISVCN53 (*bwc1*Δ+P*_GAL7_-UVE1*) grown on glucose, AISVCN53 (*bwc1*Δ+P*_GAL7_-UVE1*) grown on galactose, JEC21 (WT) 23 h dark+1 h light induction. Blots were stripped and re-probed with actin as a housekeeping gene. (B) Graphical representation of northern blot quantification. The X-axis shows gene induction in respective conditions, and Y-axis shows expression of different strains under different conditions.
(TIF)

Table S1 Primers used for gene disruption and plasmid construction.
(PDF)

Table S2 Fungal strains.
(PDF)

Table S3 Plasmids used.
(PDF)

Table S4 Primers used to amplify probes for northern blot analysis.
(PDF)
