# Supplementary figures and images for: The Uve1 Endonuclease Is Regulated by the White Collar Complex to Protect Cryptococcus neoformans from UV Damage
Source: PLoS Genet. 2013 Sep 5;9(9):e1003769. doi: 10.1371/journal.pgen.1003769 (PMC3764193; doi:10.1371/journal.pgen.1003769)

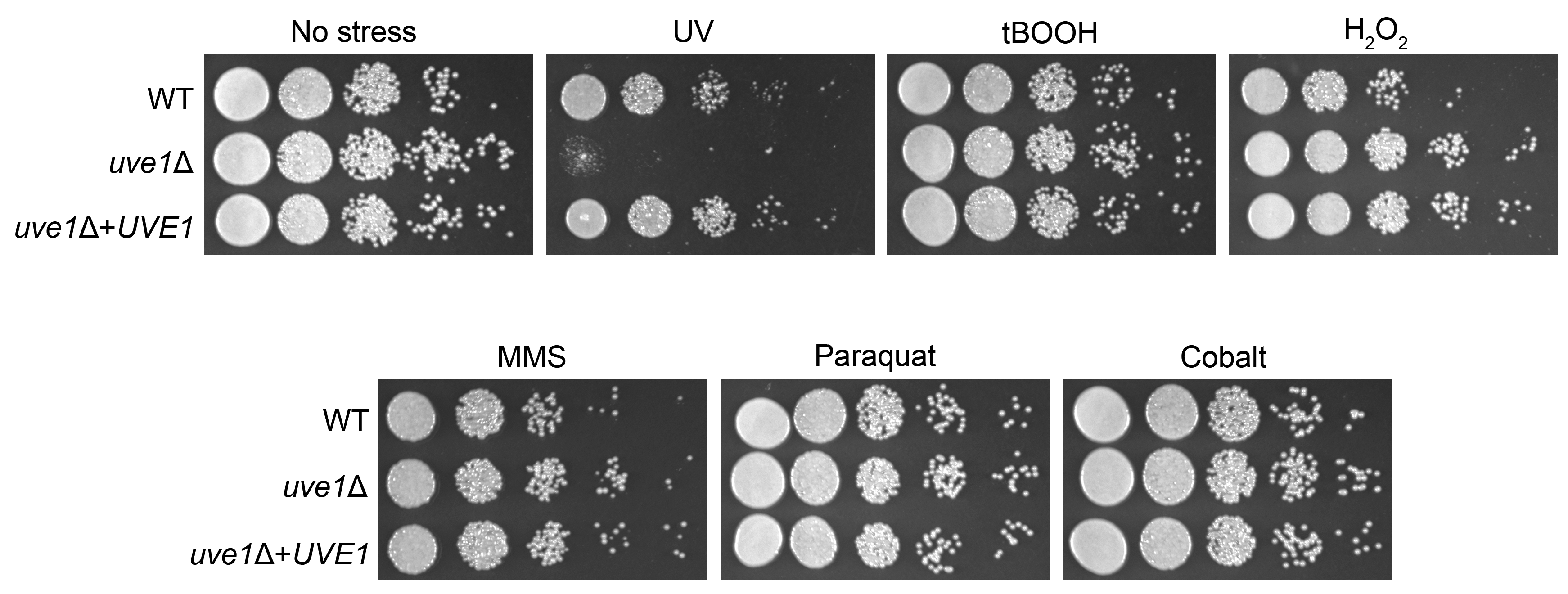

Supplement: Figure S1 — Different DNA damage chemicals, UV and heavy metal stress for the WT, uve1Δ and uve1Δ+UVE1 C. n. var. grubii strains. The order of the strains from top to bottom for each panel is KN99α (WT), AI191 (uve1Δ) and AI198 (uve1Δ+UVE1). Ten-fold serial dilutions for strains grown on YPD media at 30°C for 2 days. The stresses are UV (120 J/m2), tBOOH t-butyl hydroperoxide (0.6 mM),H2O2 hydrogen peroxide (1 mM), MMS methyl methanesulfonate (0.025%), Paraquat (0.5 mM), and cobalt chloride (60 µM). (TIF) [file pgen.1003769.s002.tif]

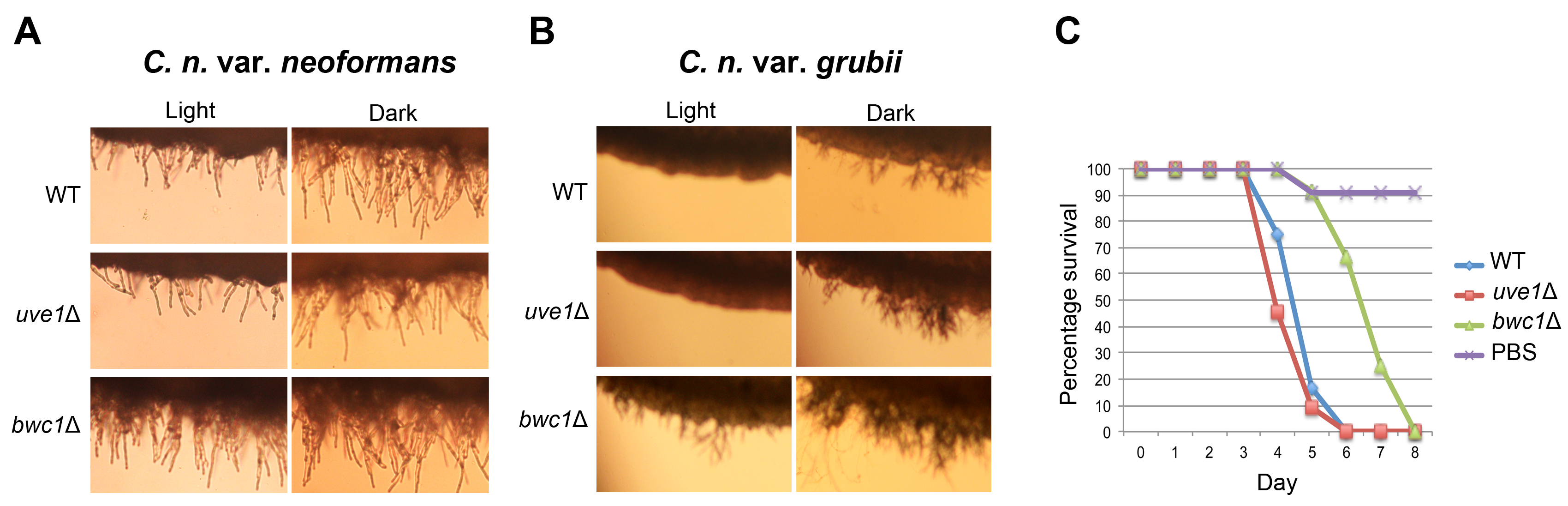

Supplement: Figure S2 — The uve1Δ strain is unaffected in mating and virulence. (A, B) Crosses were set for C. n. var. neoformans and C. n. var. grubii strains. Yeast cells of opposite mating type were mixed on V8 medium and Murashige-Skoog medium, in light grown and dark grown condition for 3 to 4 days and photographed. (A) Mating for C. n. var. neoformans, top panel JEC21 (WT, MATα) X JEC20 (WT, MAT a), middle panel AISVCN101 (uve1Δ, MATα) X AISVCN104 (uve1Δ, MAT a) and bottom panel AI5 (bwc1Δ, MATα) X AI6 (bwc1Δ, MAT a). (B) Mating for C. n. var. grubii, top panel KN99α X KN99a, middle panel AI191 (uve1Δ, MATα) X AISVCN52 (uve1Δ, MAT a), and bottom panel AI81 (bwc1Δ, MATα) X AI89 (bwc1Δ, MAT a). (C) Graphical representation for the percentage survival for wax moth larvae injected with the either PBS or 2×10^7^ cells/ml of KN99α, AI191 or AI81. (TIF) [file pgen.1003769.s003.tif]

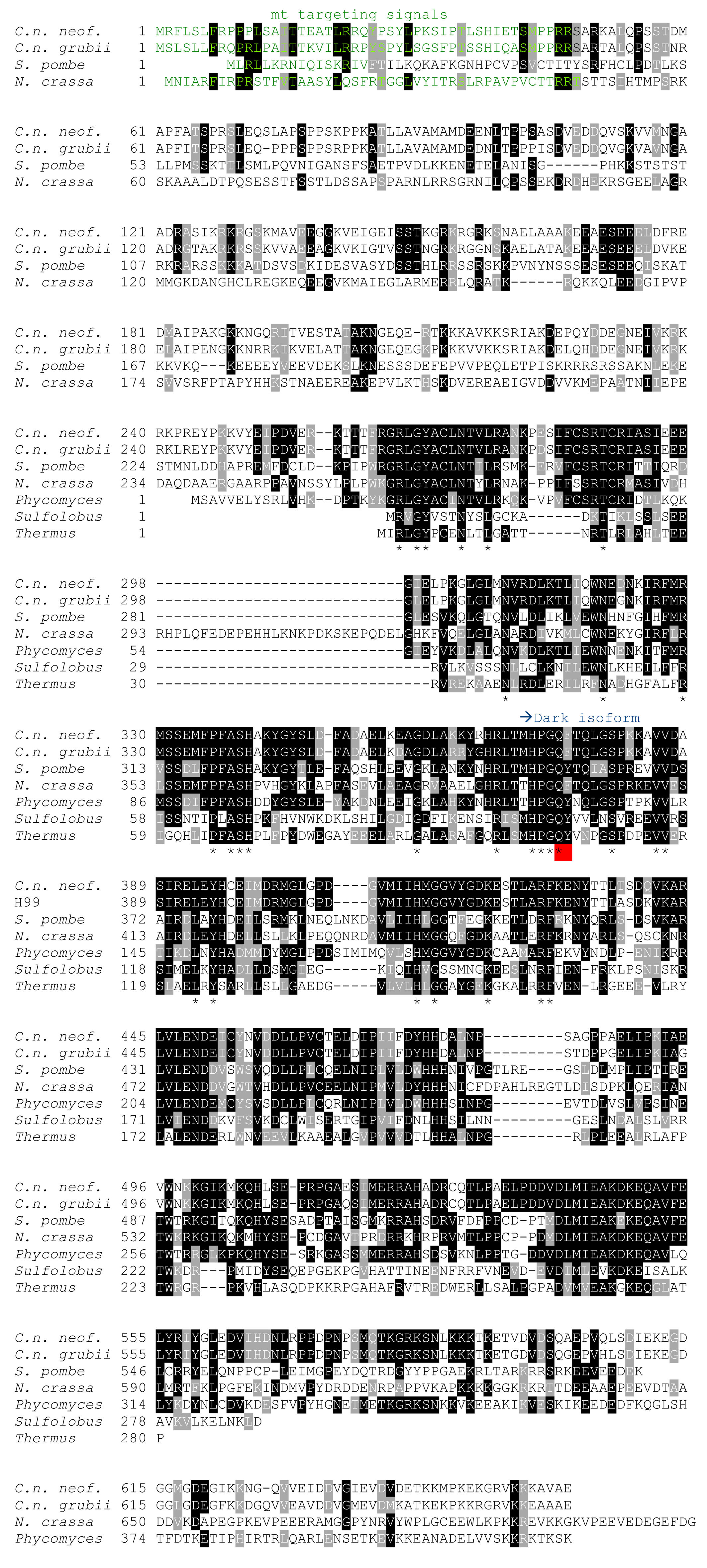

Supplement: Figure S3 — Alignment of Uve1 homologs from fungi, bacterium Thermus thermophilus and Archaea Sulfolobus acidocaldarius. Fungal species are C. neoformans var. neoformans (C. n. neof.), C. neoformans var. grubii (C. n. grubii), S. pombe, N. crassa and P. blakesleeanus. Mitochondrial localization sequences predicted by MItoProt are in green. Residues conserved in all species are marked by an asterisk. The two residues associated with the catalytic active site are highlighted in red. The start of the dark short isoform from var. neoformans is indictaed by the blue arrow. (TIF) [file pgen.1003769.s004.tif]

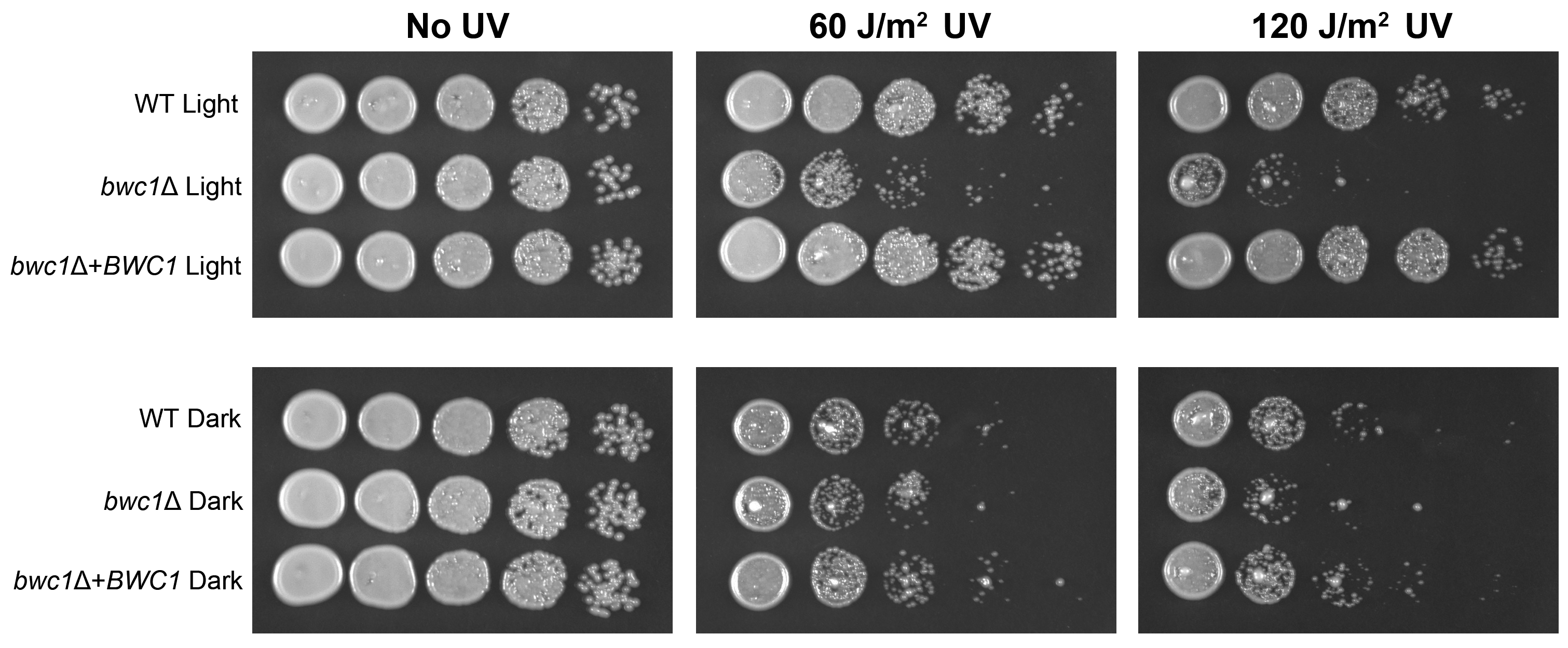

Supplement: Figure S4 — Pre-exposure to light increases resistance to UV irradiation. Ten-fold serial dilutions for strains of C. neoformans var. neoformans grown at 30°C for 2 days after UV stress. Strains used are JEC21 (WT), AI5 (bwc1Δ) and AI51 (bwc1Δ+BWC1). Top three panels are strains grown in dark and given 2 h of white light (4,400 LUX) prior to UV stress. Bottom three panels are for the strains kept in constant darkness before UV stress. (TIF) [file pgen.1003769.s005.tif]

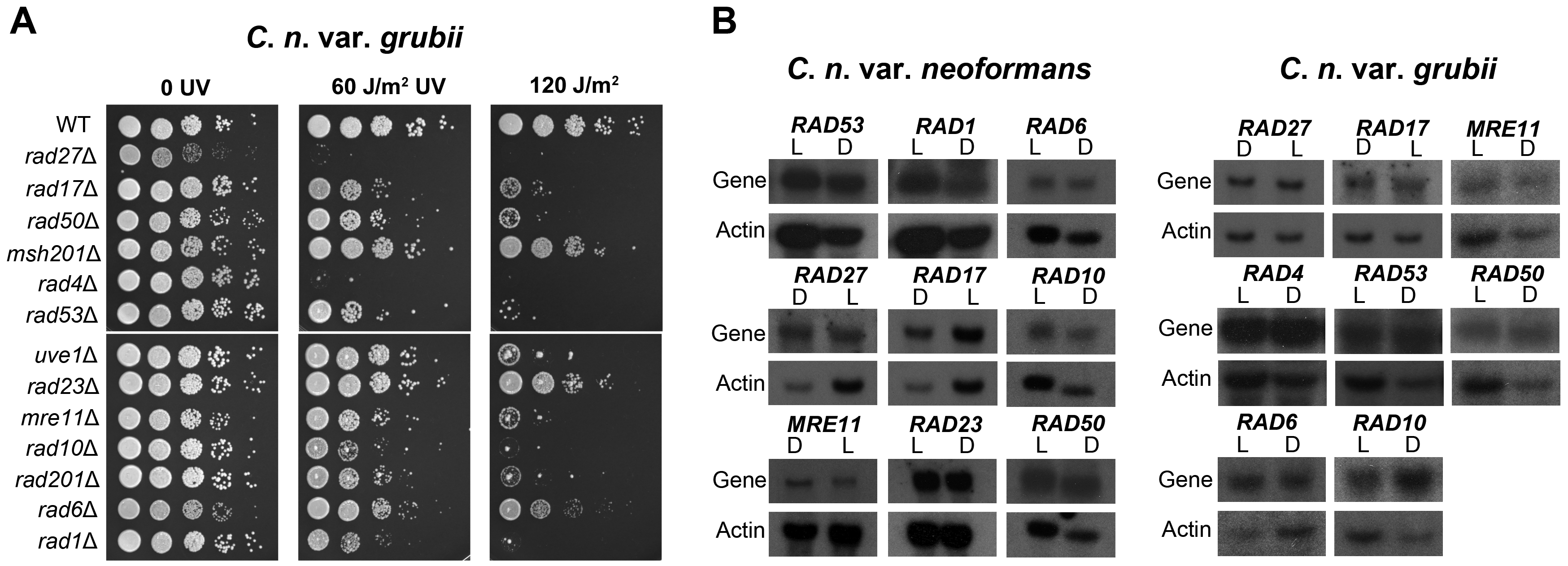

Supplement: Figure S5 — UV sensitivity of DNA repair genes and their regulation by light. (A) Ten-fold serial dilutions for C. neoformans var. grubii strains, untreated or stressed with UV and grown at 30°C for 2 days. Order of C. neoformans var. grubii strains from top to bottom is KN99α, D320 (rad27Δ), D893 (rad17Δ), D1445 (rad50Δ), D287 (msh201Δ), D397 (rad4Δ), D1053 (rad53Δ), D759 (uve1Δ), D288 (rad23Δ), D594 (mre11Δ), D1344 (rad10Δ), D203 (rad201Δ), D293 (rad6Δ), AI219 (rad1Δ). (B) Northern blots for genes implicated in repair of UV damage, in C. neoformans var. neoformans and C. neoformans var. grubii. L and D represent light grown and dark grown conditions for the strains. (TIF) [file pgen.1003769.s006.tif]

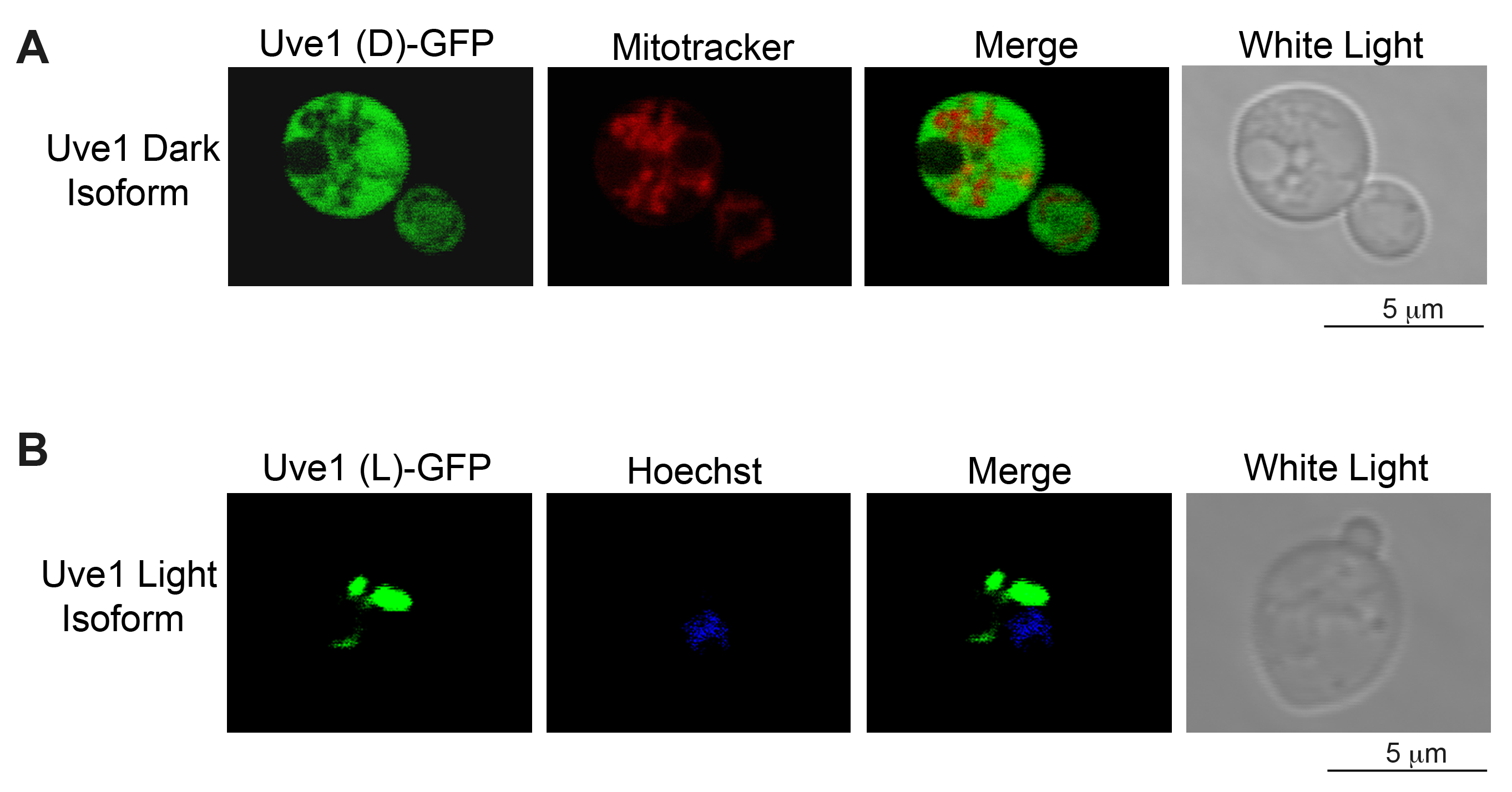

Supplement: Figure S6 — Subcellular localization of Uve1 (D)-GFP and Uve1 (L)-GFP from C. neoformans var. neoformans in C. neoformans var. grubii. (A) Uve1 (D)-GFP localization compared with MitoTracker, and (B) Uve1 (L)-GFP localization compared with nuclear Hoechst staining. The scale bar is 5 µm. (TIF) [file pgen.1003769.s007.tif]

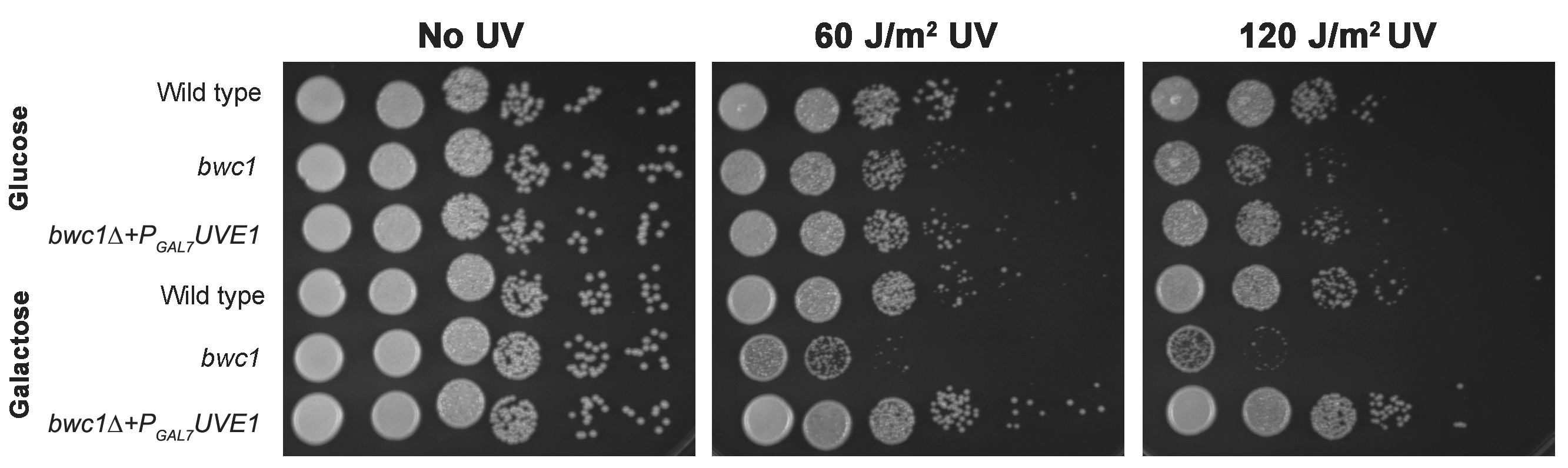

Supplement: Figure S7 — UVE1 overexpression rescues the UV sensitive phenotype of bwc1Δ mutants in C. n. var. grubii. Three strains, KN99α (WT), AI81 (bwc1Δ) and AISVCN66 (bwc1Δ+PGAL7-UVE1), were grown overnight in YNB+glucose or YNB+galactose before inoculating onto YPD plates, and grown at 30°C for 2 days. (TIF) [file pgen.1003769.s008.tif]

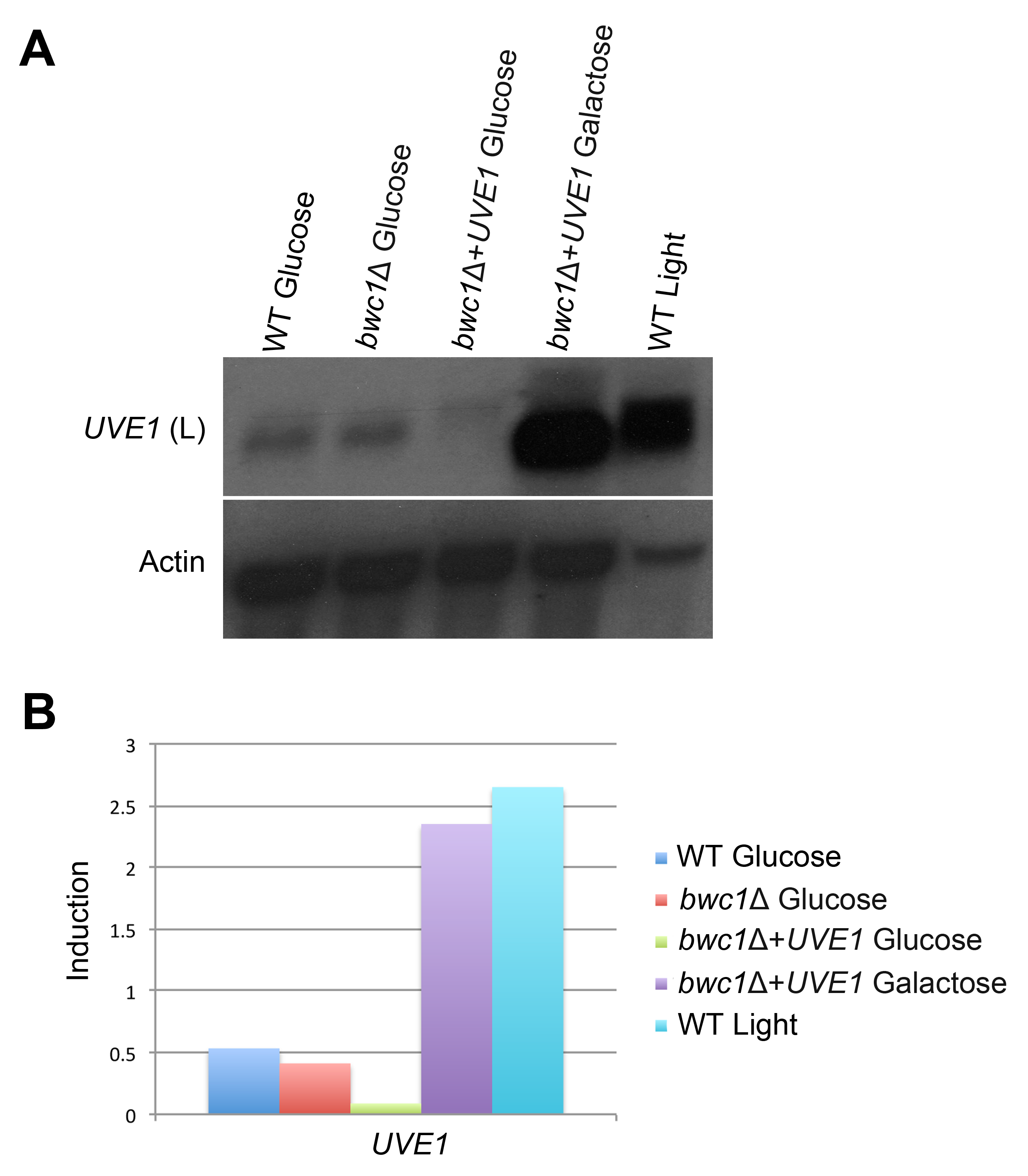

Supplement: Figure S8 — Northern blot and quantification of PGAL7-UVE1 expression in bwc1Δ compared to the level in light-induced wild type. (A) Northern blot of C. n. var. neoformans from left to right, JEC21 (WT) grown on glucose, AI5 (bwc1Δ) grown on glucose, AISVCN53 (bwc1Δ+PGAL7-UVE1) grown on glucose, AISVCN53 (bwc1Δ+PGAL7-UVE1) grown on galactose, JEC21 (WT) 23 h dark+1 h light induction. Blots were stripped and re-probed with actin as a housekeeping gene. (B) Graphical representation of northern blot quantification. The X-axis shows gene induction in respective conditions, and Y-axis shows expression of different strains under different conditions. (TIF) [file pgen.1003769.s009.tif]
