# Supplementary material for: The Uve1 Endonuclease Is Regulated by the White Collar Complex to Protect Cryptococcus neoformans from UV Damage
Source: PLoS Genet. 2013 Sep 5;9(9):e1003769. doi: 10.1371/journal.pgen.1003769 (PMC3764193; doi:10.1371/journal.pgen.1003769)
Supplement: Table S1 — Primers used for gene disruption and plasmid construction. (PDF) [file pgen.1003769.s010.pdf]

Supplemental table 1.

**Name**	**Sequence 5'-3'**	**Strain/Plasmid**	**Comments** (F forward; R reverse)
AISV001	ATGAGGTTCTTGTCGCTC	JEC21	*UVE1* (L) GFP localization (F)
AISV002	ATGCATCCAGGTCAGGTG	JEC21	*UVE1* (D) GFP localization (F)
AISV003	TTCTGCCACCGCTTTTTTCTTCAC	JEC21	*UVE1* (L/D) GFP localization (R)
AISV004	GAAAAAAGCGGTGGCAGAAATGGTGAGCAAGGGCGAG	JEC21	*UVE1* overlap primer fused to GFP (F)
AISV005	GAGCGACAAGAACCTCATGGTGATAGATGTGTTGTG	JEC21	*UVE1* (L) start fused to histone promoter
AISV006	CACCTGACCTGGATGCATGGTGATAGATGTGTTGTG	JEC21	*UVE1* (D) start fused to histone promoter
AISV007a	GCTTAGGCTATTGAAACG	L972	*S. pombe UVE1* knockout (F)
AISV007	CGTACGCTGCAGGTCGAC	pFA6a-GFP[S65ST]-kanMX6	KanMX (F)
AISV008	TCGATGAATTCGAGCTCG	pFA6a-GFP[S65ST]-kanMX6	KanMX (R)
AISV009	GTCGACCTGCAGCGTACGTTCGTGTAGACGTAGAAG	pFA6a-GFP[S65ST]-kanMX6	*S. p. UVE1-KanMx* start overlap knockout
AISV010	CGAGCTCGAATTCATCGAACAGAAGCAACATTACTC	pFA6a-GFP[S65ST]-kanMX6	*S. p. UVE1-KanMx* end overlap knockout
AISV011	CTTCTTTTCTACTACGCC	L972	For *S. pombe uve1* knockout (R)
AISV012	GTGATGATCATCCAcATGGG	cDNA JEC20	Site directed *S. p. UVE1* complementation (F)
AISV013	CCCATgTGGATGATCATCAC	cDNA JEC20	Site directed *S. p. UVE1* complementation (R)
AISV014	GGAATTCcatATGAGGTTCTTGTCGCTC	cDNA JEC20	*UVE1* (L) + NdeI for *S. p.* complementation (F)
AISV015	GGAATTCcatATGCATCCAGGTCAGTTTAC	cDNA JEC20	*UVE1* (D) + NdeI for *S. p.* complementation (F)
AISV016	CGGGATCCTGGCTATTCTGCCACCGC	cDNA JEC20	*UVE1* (L/D) + BamHI *S. p.* complementation (R)
AISV019	GTAGTCACCAGTCATTCAG	JEC21	EMSA Probe 2 *UVE1* Promoter (F)
AISV020	GAACCTCATGTACTTAATG	JEC21	EMSA Probe 2 *UVE1* Promoter (R)
AISV025	GATCAGGCGCTGGCTGTGAG	JEC21	Promoter *GAL7* (F)
AISV026	AGCGACGTCTTCTTTGCG	JEC21	*UVE1* terminator
AISV027	GCGCTCAATCCCCTCTTGAGAATGAGGTTCTTGTCGCTC	JEC21	Overlap *UVE1-GAL7* (F)
AISV028	GAGCGACAAGAACCTCATTCTCAAGAGGGGATTGAGCGC	JEC21	Overlap *UVE1-GAL7* JEC21 (R)
AISV030	CTCCACACACTCGATTC	JEC21	*UVE1* knockout (F)
AISV031	CCATCATCCCATCTCCTC	JEC21	Confirmation of *UVE1* knockout (F)
AISV032	GAAGGTGGCTTCATTCG	JEC21	*UVE1* knockout (R)
AISV033	GAGAGACTTCCGCTGGAG	JEC21	Confirmation of *UVE1* knockout (R)

AISV034	GCTTATGTGAGTCCTCCCGACTTTGCTCTAGACTTC	JEC21	*UVE1* knockout overlap with *NAT*, start
AISV035	CTCGTTTCTACATCTCTTCCAATCCCAGCATGCAGAC	JEC21	*UVE1* knockout overlap with *NAT*, end
AISV040	cgGGATCC AATGGTGCCGCCGAGTTCC	cDNA JEC21	BamHI+ Bwc2, for Bwc2 expression (F)
AISV041	cgGAATTC AAGTTGAATTTCGTTTTGCCC	cDNA JEC21	EcoRI+ Bwc2, for Bwc2 expression (R)
ai255	CATTCAGGCTGCGCAACTG	pPZP-GFP-NATcc	GFP-NAT cassette (F)
aiI256	CCAATACGCAAACCGCCTC	pPZP-GFP-NATcc	GFP-NAT cassette (R)
ai290	GGGAGGACTCACATAAGC	pJAF1/pPZP-GFP-NATcc	Neomycin or nourseothricin resistance (F)
ai006	GAAGAGATGTAGAAACGAG	pJAF1/pPZP-GFP-NATcc	Neomycin or nourseothricin resistance (R)
ALID1229	GTAACGCCAGGGTTTTCCCAGTCACGACG	pRS426	EMSA non-specific probe (F)
ALID1230	GCGGATAACAATTTCACACAGGAAACAGC	pRS426	EMSA non-specific probe (R)
ai830	GGGTTGCGTAAGTGCTGG	KN99α	*UVE1* knockout (F)
ai831	GCTTATGTGAGTCCTCCCAGGTAGTCGTGGTTGTCG	KN99α	*UVE1* knockout overlap (F)
ai832	CTCGTTTCTACATCTCTTAGCGACGAAGGAGAAGCC	KN99α	*UVE1* knockout overlap (R)
ai833	GATAGGCAGTTTCGATCC	KN99α	*UVE1* knockout (R)
ALID0001	TCTGATGCTTCTTTGGAAGG	KN99α	*UVE1* complementation (F)
ALID0002	TACCACCTTTTCCTGATGGG	KN99α	*UVE1* complementation (R)
AISV066	CCGggatccTTACTTGTACAGCTCGTC	P_{HXK2}-*RAD23-GFP*	*C. n. UVE1* (*L*)-*GFP* localization in *S. pombe*
AISV85	AGCGCTTGATTCTATGCG	KN99α	Nuclear Long / Short PCR (F)
AISV95	AGTCCTTCTCTTACTCCC	KN99α	Nuclear Long PCR (R)
AISV97	CAATTTCGCTCATGCCGT	KN99α	Nuclear Short PCR (R)
AISV87	CTAAACGGTGACCACCAAC	KN99α	Mitochondria Long PCR (F)
AISV91	GTTCAGGGGTTCGAATCCT	KN99α	Mitochondria Long PCR (R)
AISV89	CATACCAAAACCTGGAATG	KN99α	Mitochondria Short PCR (R)
AISV99	CTCCTGGTATGACACTACA	KN99α	Mitochondria Short PCR (F)
