# Supplementary material for: The Uve1 Endonuclease Is Regulated by the White Collar Complex to Protect Cryptococcus neoformans from UV Damage
Source: PLoS Genet. 2013 Sep 5;9(9):e1003769. doi: 10.1371/journal.pgen.1003769 (PMC3764193; doi:10.1371/journal.pgen.1003769)
Supplement: Table S2 — Fungal strains. (PDF) [file pgen.1003769.s011.pdf]

Supplemental table 2.

Strain	Genotype	Parents/Background	Reference
Cryptococcus			
JEC20	Wild type, *MAT***a**		[1]
JEC21	Wild type, *MAT*α		[1]
AI5	*bwc1*Δ*::URA5 ura5*, *MAT*α	JEC43	[2]
AI6	*bwc1*Δ*::URA5 ura5*, *MAT***a**	AI5 X JEC20	[2]
AI51	*bwc1*Δ*::NEO BWC1*, *MAT*α	AI5	[2]
AISVCN103	*uve1*Δ*::NAT*	JEC20	This study
AISVCN101	*uve1*Δ*::NAT*	JEC21	This study
KN99**a**	Wild type, *MAT***a**		[3]
KN99α	Wild type, *MAT*α		[3]
AI89	*bwc1*Δ*::NAT*	KN99**a**	[2]
AI81	*bwc1*Δ*::NAT*	KN99α	[2]
AI191	*uve1*Δ*::NEO*	KN99α	This study
AISVCN52	*uve1*Δ*::NEO*	KN99**a**	This study
AI198	*uve1*Δ*::NEO UVE1-NAT*	AI191	This study
ST239E6	P_{UVE1}*-T-DNA-NAT*	KN99α	[4]
AISVCN28	*uve1*Δ*::NEO UVE1* (L)-GFP	AI191	This study
AISVCN02	*uve1*Δ*::NEO UVE1* (D)-GFP	AI191	This study
VANC.R265	Wild type, *MAT*α		
AISVCN53	*bwc1*Δ*::URA5 ura5* P_{GAL7}*-UVE1-NEO*	AI5	This study
AISVCN66	*bwc1*Δ*::NAT* P_{GAL7}*-UVE1-NEO*	AI81	This study
AI219	*rad1*Δ*::NAT*	KN99α	Unpublished
D320	*rad27*Δ*::NAT*	H99	[5]
D893	*rad17*Δ*::NAT*	H99	[5]
D1445	*rad50*Δ*::NAT*	H99	[5]
D287	*msh201*Δ*::NAT*	H99	[5]
D397	*rad4*Δ*::NAT*	H99	[5]
D1053	*rad53*Δ*::NAT*	H99	[5]

D759	*uve1*Δ*::NAT*	H99	[5]
D288	*rad23*Δ*::NAT*	H99	[5]
D594	*mre11*Δ*::NAT*	H99	[5]
D1344	*rad10*Δ*::NAT*	H99	[5]
D203	*rad201*Δ*::NAT*	H99	[5]
D293	*rad6*Δ*::NAT*	H99	[5]
H99			
S. pombe			
L972	Wild type		
MM72-4A	*ura4-D18* h^{-}		
AISVSP1	*uve1*Δ::kanMX6	MM72-4A	This study
AISVSP2	*uve1*Δ::kanMX6 $ura4^{+}$	AISVSP1	This study
AISVSP3	*uve1*Δ::kanMX6 *C.n. UVE1* (D)-$ura4^{+}$	AISVSP1	This study
AISVSP4	*uve1*Δ::kanMX6 *C.n. UVE1* (L)-$ura4^{+}$	AISVSP1	This study
AISVSP15	*uve1*Δ::kanMX6 *C.n. UVE1* (L)-GFP-$ura4^{+}$	AISVSP1	This study
P. blakesleeanus			
NRRL1555	Wild type		[6]
L51	*madA madB*		[7]
N. crassa			
FGSC 4200	Wild type		[8]
FGSC 4398	*wc-1*		[9]

References

1. Kwon-Chung KJ, Edman JC, Wickes BL (1992) Genetic association of mating types and virulence in *Cryptococcus neoformans*. Infect Immun 60: 602-605.
2. Idnurm A, Heitman J (2005) Light controls growth and development via a conserved pathway in the fungal kingdom. PLoS Biol 3: e95.
3. Nielsen K, Cox GM, Wang P, Toffaletti DL, Perfect JR, et al. (2003) Sexual cycle of *Cryptococcus neoformans* var. *grubii* and virulence of congenic **a** and α isolates. Infect Immun 71: 4831-4841.
4. Idnurm A, Walton FJ, Floyd A, Reedy JL, Heitman J (2009) Identification of *ENA1* as a virulence gene of the human pathogenic fungus *Cryptococcus neoformans* through signature-tagged insertional mutagenesis. Eukaryot Cell 8: 315-326.
5. Liu OW, Chun CD, Chow ED, Chen C, Madhani HD, et al. (2008) Systematic genetic analysis of virulence in the human fungal pathogen *Cryptococcus neoformans*. Cell 135: 174-188.
6. Bergman K, Eslava AP, Cerdá-Olmedo E (1973) Mutants of *Phycomyces* with abnormal phototropism. Mol Gen Genet 123: 1-16.
7. Lipson ED, Terasaka D. T., Silverstein, P. S. (1980) Double mutants of *Phycomyces* with abnormal phototropism. Mol Gen Genet 179: 155-162.
8. Käfer E, Fraser M (1979) Isolation and genetic analysis of nuclease halo (nuh) mutants of *Neurospora crassa*. Mol Gen Genet 169: 117-127.
9. Degli-Innocenti F, Russo VE (1984) Isolation of new white collar mutants of *Neurospora crassa* and studies on their behavior in the blue light-induced formation of protoperithecia. J Bacteriol 159: 757-761.
