# Supplementary material for: The Uve1 Endonuclease Is Regulated by the White Collar Complex to Protect Cryptococcus neoformans from UV Damage
Source: PLoS Genet. 2013 Sep 5;9(9):e1003769. doi: 10.1371/journal.pgen.1003769 (PMC3764193; doi:10.1371/journal.pgen.1003769)
Supplement: Table S3 — Plasmids used. (PDF) [file pgen.1003769.s012.pdf]

Supplemental table 3.

Plasmid Name	Parent plasmid	Comments/Purpose	Reference
pFA6a-GFP[S65T]-kanMX6		*KanMX* for *S. pombe uve1* knockout	[1]
pPZP-GFP-NATcc	pPZP-NATcc	GFP-NAT cassette amplification	[2]
pJAF1		Neomycin amplification	[3]
pRS426		Non-specific probe preparation for EMSA	[4]
pREP42		*S. pombe* empty plasmid	[5]
pREP42-*UVE1*(L)	pREP42	NdeI and BamHI/*S. pombe UVE1* (L) complementation	This study
pREP42-*UVE1*(D)	pREP42	NdeI and BamHI/*S. pombe UVE1* (D) complementation	This study
pCR 2.1 TOPO-P_{GAL7}-*UVE1*	pCR 2.1 TOPO	TA cloning/*UVE1* overexpression in *bwc1*	This study
pPZP-NEO-P_{GAL7}-*UVE1*	pPZP-NEO	BamHI and XhoI/*UVE1* overexpression in *bwc1*	This study
pRSETA-*BWC2*	pRSET A	BamHI and EcoRI/Bwc2 expression	This study
pPZP-*UVE1*-NATcc	pPZP-NATcc	BamHI-SalI/*UVE1* complementation in *uve1*	This study
pTN157-*UVE1* (L)-GFP	pTN157	*S. pombe* UVE1 (L)-GFP localization	This study

References

1. Wach A, Brachat A, Alberti-Segui C, Rebischung C, Philippsen P (1997) Heterologous *HIS3* marker and GFP reporter modules for PCR-targeting in *Saccharomyces cerevisiae*. Yeast 13: 1065-1075.
2. Idnurm A, Giles SS, Perfect JR, Heitman J (2007) Peroxisome function regulates growth on glucose in the basidiomycete fungus *Cryptococcus neoformans*. Eukaryot Cell 6: 60-72.
3. Fraser JA, Subaran RL, Nichols CB, Heitman J (2003) Recapitulation of the sexual cycle of the primary fungal pathogen *Cryptococcus neoformans* var. *gattii*: implications for an outbreak on Vancouver Island, Canada. Eukaryot Cell 2: 1036-1045.
4. Colot HV, Park G, Turner GE, Ringelberg C, Crew CM, et al. (2006) A high-throughput gene knockout procedure for *Neurospora* reveals functions for multiple transcription factors. Proc Natl Acad Sci U S A 103: 10352-10357.
5. Basi G, Schmid E, Maundrell K (1993) TATA box mutations in the *Schizosaccharomyces pombe nmt1* promoter affect transcription efficiency but not the transcription start point or thiamine repressibility. Gene 123: 131-136.
