# Supplementary material for: The Uve1 Endonuclease Is Regulated by the White Collar Complex to Protect Cryptococcus neoformans from UV Damage
Source: PLoS Genet. 2013 Sep 5;9(9):e1003769. doi: 10.1371/journal.pgen.1003769 (PMC3764193; doi:10.1371/journal.pgen.1003769)
Supplement: Table S4 — Primers used to amplify probes for northern blot analysis. (PDF) [file pgen.1003769.s013.pdf]

Supplemental table 4.

Primer	Sequence 5'-3'	Strain	Gene	Organism
AISV001	ATGAGGTTCTTGTCGCTC	JEC21	*UVE1*	*C. n.* var. *neoformans*
AISV003	TTCTGCCACCGCTTTTTTCTTCAC	JEC21	*UVE1*	*C. n.* var. *neoformans*
AISV055	ATGCCTCCCCGAAGAAGTG	KN99α	*UVE1*	*C. n.* var. *grubii*
AISV056	CTCTGCCGCCGCTTCTTTC	KN99α	*UVE1*	*C. n.* var. *grubii*
AISV043	GAAGAAGTGCACGTACAG	R265	*UVE1*	*C. gattii*
AISV0044	CTAGGGCATTTCTTTCGT	R265	*UVE1*	*C. gattii*
AISV007a	GCTTAGGCTATTGAAACG	L972	*UVE1*	*S. pombe*
AISV011	CTTCTTTTCTACTACGCC	L972	*UVE1*	*S. pombe*
ALID1415	TAAACTCGATTATACGGTCC	FGSC 4200	*UVE1*	*N. crassa*
ALID1442	GCTTATGTGAGTCCTCCCATATACTAGGAGCTAGCC	FGSC 4200	*UVE1*	*N. crassa*
ALID1412	GCTTAGTACACAAAGATCCG	NRRL1555	*UVE1*	*P. blakesleeanus*
ALID1413	GATACTACTAGCTCATCTGC	NRRL1555	*UVE1*	*P. blakesleeanus*
ai018	ATGGAAGAAGAAGGTACG	JEC21	Actin	*C. n.* var. *neoformans*
ai019	TTAGAAACACTTTCGGTG	JEC21	Actin	*C. n.* var. *neoformans*
ai018	ATGGAAGAAGAAGGTACG	KN99α	Actin	*C. n.* var. *grubii*
ai019	TTAGAAACACTTTCGGTG	KN99α	Actin	*C. n.* var. *grubii*
AISV045	ATGTCTATGGAAGAAGAAG	R265	Actin	*C. gattii*
AISV046	ACCAGACTCGTCGTACTCG	R265	Actin	*C. gattii*
ALID0752	AAGAAATCGCAGCGTTGG	L972	Actin	*S. pombe*
ALID0753	CACTTACGGTAAACGATACC	L972	Actin	*S. pombe*
ai1677	TCTACTGGTTATCATGGG	FGSC 4200	Actin	*N. crassa*
ai1678	AGAAGCACTTGCGGTGCACG	FGSC 4200	Actin	*N. crassa*
ai679	CACACTTTCTACAACGAG	NRRL1555	Actin	*P. blakesleeanus*
ai680	ACATCTGCTGGAAGGTAG	NRRL1555	Actin	*P. blakesleeanus*
AISV69	ACAGTGACGATGTCTTTG	JEC21	*RAD53*	*C. n.* var. *neoformans*
AISV70	AGGCGCATAGATTTTCGG	JEC21	*RAD53*	*C. n.* var. *neoformans*
AISV69	ACAGTGACGATGTCTTTG	KN99α	*RAD53*	*C. n.* var. *grubii*

AISV70	AGGCGCATAGATTTTCGG	KN99α	*RAD53*	*C. n.* var. *grubii*
AISV71	TGACATGCCGCCCAATAC	JEC21	*RAD50*	*C. n.* var. *neoformans*
AISV72	GCGCATTGATATTCTCTTG	JEC21	*RAD50*	*C. n.* var. *neoformans*
AISV71	TGACATGCCGCCCAATAC	KN99α	*RAD50*	*C. n. var. grubii*
AISV72	GCGCATTGATATTCTCTTG	KN99α	*RAD50*	*C. n.* var. *grubii*
AISV73	CAAGCGACAGGAGCCTCA	JEC21	*RAD17*	*C. n.* var. *neoformans*
AISV74	CGTCCACCAAATAATCCTC	JEC21	*RAD17*	*C. n.* var. *neoformans*
AISV73	CAAGCGACAGGAGCCTCA	KN99α	*RAD17*	*C. n.* var. *grubii*
AISV74	CGTCCACCAAATAATCCTC	KN99α	*RAD17*	*C. n.* var. *grubii*
AISV75	ACCCGACTCACAACCAAG	JEC21	*MRE11*	*C. n.* var. *neoformans*
AISV76	CTAATCCTCATCTGATGAC	JEC21	*MRE11*	*C. n.* var. *neoformans*
AISV75	ACCCGACTCACAACCAAG	KN99α	*MRE11*	*C. n.* var. *grubii*
AISV76	CTAATCCTCATCTGATGAC	KN99α	*MRE11*	*C. n.* var. *grubii*
AISV77	ATGACTGAGCAGAACAG	JEC21	*RAD10*	*C. n.* var. *neoformans*
AISV78	CACCTTGTCATCATCCT	JEC21	*RAD10*	*C. n.* var. *neoformans*
AISV77	ATGACTGAGCAGAACAG	KN99α	*RAD10*	*C. n.* var. *grubii*
AISV78	CACCTTGTCATCATCCT	KN99α	*RAD10*	*C. n.* var. *grubii*
AISV81	GCTTGAGATCGATGCTG	JEC21	*RAD6*	*C. n.* var. *neoformans*
AISV82	TCCGCTTCAACCACTTC	JEC21	*RAD6*	*C. n.* var. *neoformans*
AISV81	GCTTGAGATCGATGCTG	KN99α	*RAD6*	*C. n.* var. *grubii*
AISV82	TCCGCTTCAACCACTTC	KN99α	*RAD6*	*C. n.* var. *grubii*
AISV83	GACACATCCCTTATCTTCC	JEC21	*RAD1*	*C. n.* var. *neoformans*
AISV84	GATGTAGAAACTCCCAAGC	JEC21	*RAD1*	*C. n.* var. *neoformans*
SV16RAD27F	ATGGGTATTAAAGGTGAG	JEC21	*RAD27*	*C. n.* var. *neoformans*
SV16RSD27R	CTTCTTATTCTTCTTCTTCG	JEC21	*RAD27*	*C. n.* var. *neoformans*
SV16RAD27F	ATGGGTATTAAAGGTGAG	KN99α	*RAD27*	*C. n.* var. *grubii*
SV16RSD27R	CTTCTTATTCTTCTTCTTCG	KN99α	*RAD27*	*C. n.* var. *grubii*
SV20RAD4F	ATGAGCGCTTCCAGACC	KN99α	*RAD4*	*C. n.* var. *grubii*
SV21RAD4R	AGTCTGATTAATTTTATCTTCC	KN99α	*RAD4*	*C. n.* var. *grubii*
SV24RAD23NcoIF	CATGCCATGGTCAAGATCACTTTC	JEC21	*RAD23*	*C. n.* var. *neoformans*
SV25RAD23NcoIR	CATGCCATGGGTTGATCCTCCTCCATG	JEC21	*RAD23*	*C. n.* var. *neoformans*
